# Combating the Coronavirus Pandemic: Early Detection, Medical Treatment, and a Concerted Effort by the Global Community

**DOI:** 10.34133/2020/6925296

**Published:** 2020-06-16

**Authors:** Zichao Luo, Melgious Jin Yan Ang, Siew Yin Chan, Zhigao Yi, Yi Yiing Goh, Shuangqian Yan, Jun Tao, Kai Liu, Xiaosong Li, Hongjie Zhang, Wei Huang, Xiaogang Liu

**Affiliations:** ^1^Department of Chemistry, National University of Singapore, Singapore 117543, Singapore; ^2^NUS Graduate School for Integrative Sciences and Engineering, Singapore 117456, Singapore; ^3^Frontiers Science Center for Flexible Electronics & Shaanxi Institute of Flexible Electronics, Northwestern Polytechnical University, Xi'an 710072, China; ^4^Sports Medical Centre, The Second Affiliated Hospital of Nanchang University, Nanchang 330000, China; ^5^State Key Laboratory of Rare Earth Resource Utilization, Chang Chun Institute of Applied Chemistry, Chinese Academy of Sciences, Changchun 130022, China; ^6^Department of Oncology, The Fourth Medical Center of Chinese People's Liberation Army General Hospital, Beijing 100048, China; ^7^Department of Chemistry, Tsinghua University, Beijing 100084, China; ^8^Key Laboratory of Flexible Electronics & Institute of Advanced Materials, Nanjing Tech University, Nanjing 211816, China; ^9^The N.1 Institute for Health, National University of Singapore, Singapore; ^10^Joint School of National University of Singapore and Tianjin University, International Campus of Tianjin University, Fuzhou 350807, China

## Abstract

The World Health Organization (WHO) has declared the outbreak of 2019 novel coronavirus, known as 2019-nCoV, a pandemic, as the coronavirus has now infected over 2.6 million people globally and caused more than 185,000 fatalities as of April 23, 2020. Coronavirus disease 2019 (COVID-19) causes a respiratory illness with symptoms such as dry cough, fever, sudden loss of smell, and, in more severe cases, difficulty breathing. To date, there is no specific vaccine or treatment proven effective against this viral disease. Early and accurate diagnosis of COVID-19 is thus critical to curbing its spread and improving health outcomes. Reverse transcription-polymerase chain reaction (RT-PCR) is commonly used to detect the presence of COVID-19. Other techniques, such as recombinase polymerase amplification (RPA), loop-mediated isothermal amplification (LAMP), clustered regularly interspaced short palindromic repeats (CRISPR), and microfluidics, have allowed better disease diagnosis. Here, as part of the effort to expand screening capacity, we review advances and challenges in the rapid detection of COVID-19 by targeting nucleic acids, antigens, or antibodies. We also summarize potential treatments and vaccines against COVID-19 and discuss ongoing clinical trials of interventions to reduce viral progression.

## 1. Introduction

The recent global outbreak of COVID-19 has led to a public health emergency. As of April 23, 2020, over 2.6 million confirmed cases were reported to WHO from 213 countries and territories [1]. On January 30, 2020, WHO declared the COVID-19 outbreak as the sixth public health emergency of international concern, following H1N1 (2009), Polio (2014), Ebola in West Africa (2014), Zika (2016), and Ebola (2019) [2]. The rapid global expansion and rising fatalities have raised grave concerns on the viral spread across the globe. With the rapid increase in the number of confirmed cases, WHO classified the global COVID-19 outbreak as a pandemic on March 11, 2020 [3]. COVID-19 can spread from person-to-person and animal, and transmission of infection may occur with exposure to symptomatic patients or asymptomatic individuals.

**Figure 1:**
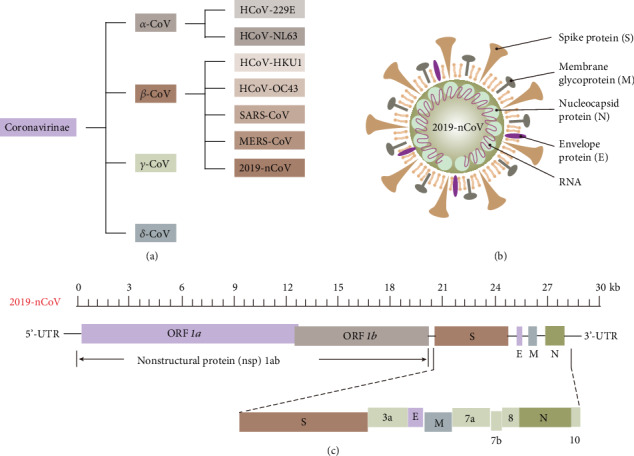
Biological and genomic structure of 2019-nCoV. (a) Classification of coronavirus genera. (b) Schematic structure of 2019-nCoV. (c) The whole-genome structure of 2019-nCoV.

Coronaviruses (CoVs) (corona: crown-like shape) are enveloped, single-stranded RNA viruses that belong to the order *Nidovirales* in the subfamily *Coronaviridae*. CoVs are divided into four genera: alpha (*α*), beta (*β*), gamma (*γ*), and delta (*δ*) (Figure 1(a)) [4]. Alpha- and beta-CoVs infect mammals, while gamma- and delta-CoVs primarily infect birds [5]. Before December 2019, six types of CoVs had infected humans, including two *α*-CoVs (HCoV-229E and HCoV-NL63) and four *β*-CoVs (HCoV-OC43, HCoV-HKU1, SARS-CoV, and MERS-CoV). The first two *β*-CoVs (HCoV-OC43 and HCoV-HKU1) mainly cause self-limiting upper respiratory infections, while the other two *β*-CoVs (SARS-CoV and MERS-CoV) are mostly associated with severe respiratory illness [6, 7]. Full-genome sequence analysis of 2019-nCoV confirms that it is a *β*-CoV, distinct from SARS-CoV and MERS-CoV [8]. Investigations reveal that 2019-nCoV shares ~80% sequence identity with SARS-CoV while maintaining ~89% nucleotide identity to the SARS-like CoVs (ZC45 and ZXC21) from bats [9]. A recent report suggests that a bat CoV (RatG13) is 96% identical to 2019-nCoV [10].

A typical CoV genome is a single-stranded, positive-sense RNA (+ssRNA) (~30 kb) enclosed by a 5′-cap and 3′-poly-A tail [11]. The genome size of 2019-nCoV is 29,891 nucleotides, encoding 9860 amino acids, with a G+C content of 38% [12]. The 2019-nCoV genome contains two flanking untranslated regions (UTRs) on 5′- and 3′-terminals, one single long open reading frame *1ab* (ORF*1ab*) encoding a polyprotein and at least five other ORFs encoding structural proteins, and eight accessory proteins (Figure 1(c)). The first ORF (ORF*1a/b*) is about two-thirds of the whole-genome length and encodes the 16 nonstructural proteins (nsp1-16). The other one-third of the genome contains four ORFs encoding the spike (S), membrane (M), envelope (E), and nucleocapsid (N) proteins, whereas other ORFs encode accessory proteins (Figure 1(b) and (c)). Most of the nonstructural proteins are essential for 2019-nCoV replication, while structural proteins are responsible for virion assembly and viral infection [12, 13]. The M and E proteins are required in viral assembly, while the N protein involves RNA genome assembly.

The S protein, a surface-located trimeric glycoprotein of CoVs, is the primary determinant of CoV tropism, as it binds to the membrane receptor on host cells, mediating viral and cellular membrane fusion [14]. The S protein of 2019-nCoV reportedly binds to angiotensin-converting enzyme 2 (ACE2), a homolog of ACE on host cell membranes, contributing to 2019-nCoV cell invasion [15]. Moreover, this particular S protein shows a higher binding affinity to ACE2 than the S protein of SARS-CoV, enabling 2019-nCoV to invade host cells more effectively [16, 17]. Recently, a transmembrane glycoprotein, CD147, also known as Basigin or EMMPRIN, has been confirmed as another receptor for binding of the 2019-nCoV S protein, thereby mediating viral invasion [18].

The E protein is an integral membrane protein that regulates viral life cycles, including pathogenesis, envelope formation, assembly, and budding [19–21]. Among the four structural proteins, protein E appears to have the highest antigenicity and the most significant potential as an immunogenic target, highlighting the possibility of developing protein E-derived peptides as a 2019-nCoV vaccine [22]. Systemic studies of proteins S and E have inspired scientists to take creative approaches to design anti-COVID-19 drugs.

Although some COVID-19 patients show no symptoms, most patients have some common symptoms such as fever, cough, fatigue, sputum production, shortness of breath, sore throat, and headache. In some severe cases, infections can cause pneumonia, severe acute respiratory syndrome, kidney failure, and death. According to the WHO-China joint report [23], on average, people infected with 2019-nCoV develop mild respiratory symptoms and fever, 5-6 days after infection (mean incubation period, 5-6 days; range, 1-14 days). People over 60 years of age and those with hypertension, diabetes, or cardiovascular diseases are at high risk for severe illness and death. In comparison, children under 19 years appear to be infected minimally by 2019-nCoV (around 2.4% of all reported cases). Based on the Chinese Center for Disease Control and Prevention (China CDC) report (from 72,314 patient records, dated 11 February 2020), among the confirmed cases, 86.6% of patients are 30-79 years of age, 80.9% of patients have mild-to-moderate disease, 13.8% have a severe illness, and 6.1% are critically ill [24]. Notably, the mortality rate of children under 19 years is 0.2%, while people aged over 80 years have the highest mortality rate of 14.8%.

Currently, there are no effective antiviral drugs or specific vaccines against COVID-19. Thus, there is an urgent need for rapid detection to prevent further spread, to reduce the intensity of the pandemic, and to slow the increase in cases. Recently, several new technologies, including LAMP-LFA, RPA-LFA, RPA-CRISPR, and other nanomaterial-based IgG/IgM kits, have been adopted for 2019-nCoV detection. A significant number of drug candidates, including chemical drugs, biological drugs, nutritional interventions, and traditional Chinese medicine (TCM), have been proposed for clinical trials after the 2019-nCoV outbreak. In this review, we concentrate on the most significant developments in 2019-nCoV detection and provide an overview of medical treatments and vaccines currently in development to combat and contain the disease.

## 2. 2019-nCoV Detection

According to the *Diagnosis and Treatment Guidelines for COVID*-*19* (7^th^ edition), COVID-19 cases can be divided into suspected cases and confirmed cases [25]. Diagnostic methods for 2019-nCoV are determined by the intrinsic properties of the virus and biomarkers that hosts exhibit after infection. These biomarkers include viral proteins and nucleic acids, as well as antibodies induced in response to viral infection. The most common 2019-nCoV detection methods include viral nucleic acid detection and serum antibody (IgG or IgM) detection. A confirmed case should have at least one of the following criteria: (i) a positive result for 2019-nCoV nucleic acid, using real-time PCR tests from respiratory or blood samples; (ii) a high homogeneity between viral gene sequencing from respiratory or blood samples and known 2019-nCoV; and (iii) serum samples positive for IgM or IgG to 2019-nCoV, or seroconversion in IgG, or a fourfold or more significant increase in IgG antibody titer to 2019-nCoV in the recovery phase than in the acute phase [25].

### 2.1. Nucleic Acid Targeting

#### 2.1.1. High-Throughput Sequencing (2^nd^-Generation Sequencing)

**Figure 2:**
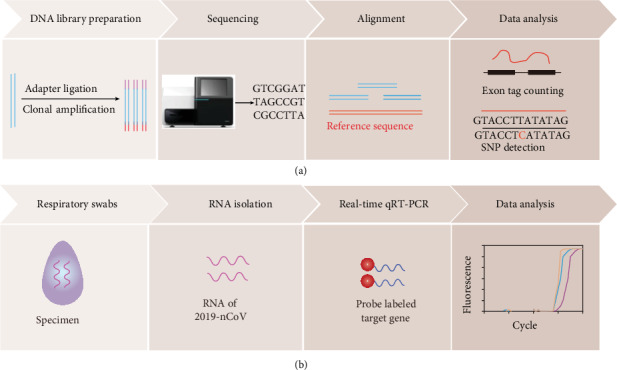
High-throughput sequencing and real-time qRT-PCR-based detection of 2019-nCoV. (a) Four steps of high-throughput sequencing technology. (b) Steps for real-time RT-PCR analysis.

High-throughput sequencing (HTS) technology contains various strategies that depend on a combination of library preparation, sequencing and mapping, genome alignment, and data analysis [26] (Figure 2(a)). Unlike the 1977 Sanger sequencing method (1^st^-generation sequencing) [27], 2^nd^-generation sequencing has been widely applied in genome sequencing, transcriptional profiling (RNA-seq) disease mapping, and population genetic studies. The whole-genome nucleotide sequence of 2019-nCoV was identified and compared with the full-length genome sequence of coronavirus from bats [10] through HTS. HTS-based technology is also applied to detect 2019-nCoV. For example, Wang et al. developed a HTS method based on nanopore target sequencing (NTS) by harnessing the benefits of target amplification and long-reads for real-time nanopore sequencing [28].

This NTS strategy detects 2019-nCoV with higher sensitivity (100-fold) than standard qPCR, simultaneously with other respiratory viruses within 6-10 h. Moreover, all targeted regions can be identified by NTS in higher copies of samples (1000-3000 copies/mL) within 10 min, indicating the potential for rapid detection of an outbreak in the clinic. For 1 h sequencing data, reads mapped to 2019-nCoV differed remarkably from those of negative controls in all targeted regions at concentrations ranging from 10 to 3000 copies/mL. Importantly, NTS can identify mutated nucleic acids. However, the NTS platform cannot readily detect highly degraded nucleic acid fragments that are less than 200 base pairs in length [29]. Moreover, the strategy requires tedious sample preparation and lengthy turnaround time.

Although HTS technology provides fast, low-cost DNA sequencing, it is not suitable for detection in clinics. On the other hand, the HTS strategy may be suitable for amplicon sequencing or de novo sequencing of a whole genome [30].

#### 2.1.2. Real-Time Reverse Transcription-Polymerase Chain Reaction (RT-PCR)

RT-PCR is considered the gold standard to detect nucleic acids extracted from 2019-nCoV specimens qualitatively. Positive results indicate infection with 2019-nCoV. RT-PCR is an advanced technique for coronavirus detection because of its optimized sensitivity, specificity, and simplicity for quantitative assay [31, 32]. It provides accurate and reliable identification for confirmed and suspected cases. There are many commercial 2019-nCoV detecting kits with oligonucleotide primers and probes (SYBR Green or TaqMan chemistries) for detecting double genes of 2019-nCoV (nucleocapsid N gene and ORF*1ab*/E/ORF*1b*/S gene). This strategy usually requires four steps: sample collection (respiratory swabs), sample preparation (RNA isolation), one-step qRT-PCR, and data analysis (Figure 2(b)) [33]. The evaluation procedure typically lasts 4-6 h. Recently, Roche (Indiana, USA) developed two automated cobas® 2019-nCoV test systems: cobas® 6800 and cobas® 8800 (approved in testing patient samples by the US FDA), which can process up to 384 results and 1056 results in an 8-hour shift, respectively [34]. The tests produce results in about 3.5 h and can process up to 4128 results in 24 h, boosting screening capacity to help restrain the sudden growing epidemic in the USA. RT-PCR is widely applicable to 2019-nCoV detection in the clinic, but limitations of this technology are obvious, such as high false-negative rate and low sensitivity. False-negative results may occur due to the following factors: first, mutations in the primers and probe-target regions in the 2019-nCoV genome [32]; second, low viral load present in test specimens, improper extraction of nucleic acid from clinical samples, or inappropriate restrictions on sample collection, transportation, or handling [31]. Real-time RT-PCR has been adopted as the gold standard diagnostic approach for 2019-nCoV worldwide. However, RT-PCR is time-consuming (4-6 h) and requires well-equipped laboratories and skilled technicians, thereby limiting full deployment in developing countries.

#### 2.1.3. Reverse Transcription- Isothermal Amplification (RT-IAMP)-Based Detection

Isothermal amplification technology has been developed to eliminate the need for a high-precision instrument in RT-PCR assays. This approach can amplify DNA at isothermal conditions without a thermocycler [35]. There are mainly four isothermal amplification technologies for nucleic acid detection: LAMP, RPA, nucleic acid sequence-based amplification (NASBA), and transcription-mediated amplification (TMA) [36]. In NASBA and TMA assays, input RNA is converted to a double-stranded DNA intermediate with a promoter region. Detection of RNA using DNA polymerase-based amplification requires a reverse transcriptase step. LAMP and RPA do not require thermal or chemical melting with the aid of enzymes. Combined with a visual detection platform, such as a lateral flow assay (LFA) or organic dyes, LAMP and RPA have been widely employed in viral detection kits.

**Figure 3:**
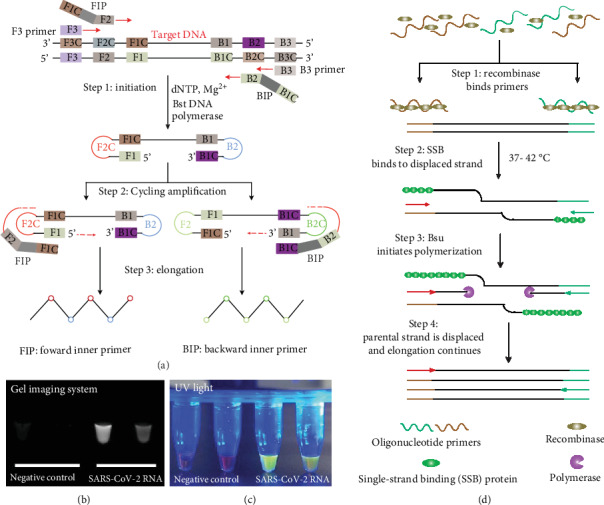
Isothermal amplification of nucleic acids for detecting 2019-nCoV. (a) The mechanism and process of loop-mediated isothermal amplification technology. (b, c) RT-LAMP combination with SYBR Green for detection of 2019-nCoV. The signal of SYBR Green dye was detected with a gel imaging system (b) and was visible with the naked eye under blue light (c). (d) The mechanism and process of recombinase polymerase amplification technology. Adapted and copyright with permission (b, c) [42], medRxiv.

LAMP is a rapid, one-step amplification technique that amplifies nucleic acids with high sensitivity and specificity at an optimal temperature of 65°C [37]. LAMP processing comprises three steps: an initial step, a cycling amplification step, and an elongation step (Figure 3(a)). LAMP employs six primers to amplify targeted genes by creating stem-loop structures that promote new DNA synthesis using a DNA polymerase with strand displacement activity. The two inner primers (FIP, BIP) and two outer primers (F3, B3), along with loop structures (LF, LB), create multiple initiating sites in the growing DNA products, enabling rapid amplification. LAMP is also highly specific since the amplification reaction occurs only when the primers correctly recognize all six regions. A reverse transcriptase step is included in the LAMP reaction to allow RNA targets to be detected [38].

RT-LAMP offers improved sensitivity and specificity in screening SARS-CoV, HCoV-NL63, and MERS-CoV compared to conventional real-time RT-PCR [39–41]. Recently, Yu et al. used a commercial LAMP kit to amplify fragmented ORF*1ab* genes of 2019-nCoV (Figure 3(b) and (c)) [42]. They optimized the LAMP system through incubation at 65°C for different periods using a 2019-nCoV-positive RNA sample as the template. Results require a 15 min reaction time at 65°C, and detection sensitivity is comparable to that of the TaqMan-based qPCR approach (10 copies). RT-LAMP employs two additional protocols for 2019-nCoV RNA detection. Park et al. performed RT-LAMP at 65°C for 40 min to identify the nsp3, S, and N genes of 2019-nCoV using colorimetric detection [43]. The sensitivity of this RT-LAMP assay was 100 copies of 2019-nCoV RNA. The other RT-LAMP protocol was conducted at 63°C for 30 min to detect the ORF*1ab*, E, and N genes simultaneously [44]. The results confirmed the specific nature of ORF*1ab* and the high sensitivity of the N gene. Based on an analysis of 208 clinical specimens, the sensitivity of this RT-LAMP was similar to conventional RT-PCR, and the specificity was 99%. Interestingly, EI-Tholoth et al. designed a two-stage isothermal amplification procedure by combining RPA (37°C) with LAMP (63°C) to detect synthesized DNA fragments of 2019-nCoV [45]. The test was performed in closed tubes within 1 h using either fluorescence or colorimetric detection. This method has a sensitivity of 100 times better than conventional LAMP and RT-PCR, suggesting a rapid, sensitive, point-of-care test for use at home.

RPA is an isothermal DNA amplification method that utilizes a specific combination of enzymes and proteins (recombinase, single-strand binding (SSB) protein, and strand-displacing DNA polymerase) to amplify target genes rapidly at a constant low temperature between 25 and 42°C in as little as 15 min [46]. RPA usually requires four steps to achieve DNA amplification: formation of a recombinase-primer complex, strand invasion, D-loop formation (stabilized by SSB, DNA polymerization through the use of strand-displacing DNA polymerase), and DNA amplification (Figure 3(d)) [47]. Results of RPA can be detected by agarose gel electrophoresis, quantitatively measured using TwistAmp^™^ probes, or simply applied in lateral flow assays. Apart from DNA target amplification, RPA formats have been developed for the detection of RNA targets (RT-RPA) by adding a reverse transcriptase enzyme to reaction mixtures [48]. Because RPA- (RT-RPA-) based detection achieves more rapid and sensitive results and operates efficiently, it has been widely adopted to detect animal and human pathogens, such as hand, foot, and mouth disease (HFMD) virus, human immunodeficiency virus (HIV), bovine coronavirus, or MERS-CoV [49–52]. Currently, RPA has been applied to detect 2019-nCoV, in combination with other technologies, such as CRISPR or microfluidic technology.

#### 2.1.4. Clustered Regularly Interspaced Short Palindromic Repeat- (CRISPR-) Based Detection

The CRISPR-associated protein 9 (Cas9) system (CRISPR/Cas9) is a revolutionary gene-editing toolbox that can modify target genes with high precision and that can control various types of genetic diseases in preclinical studies [53–56]. Due to the collateral nucleic acid cleavage activity of Cas effectors, CRISPR/Cas systems have also been widely used in nucleic acid detection with fluorescent and colorimetric signals [56]. There are mainly two kinds of CRISPR/Cas systems for diagnostics, based on the cutting activity of Cas protein on nucleic acids outside of the gRNA target site: the CRISPR/Cas13a and CRISPR/Cas12a systems.

**Figure 4:**
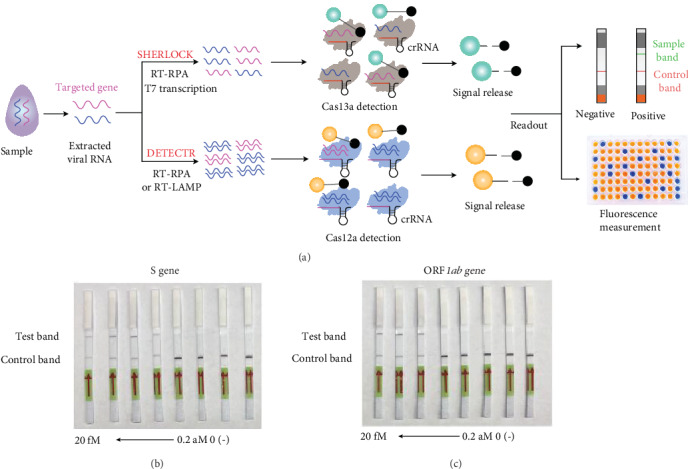
CRISPR-Cas system-based detection of 2019-nCoV. (a) Mechanism of SHERLOCK and DETECTR for 2019-nCoV detection. (b, c) Typical images of lateral flow readout for CRISPR-based detection at various concentrations [60].

The CRISPR/Cas13a system (specific high-sensitivity enzymatic reporter unlocking (SHERLOCK)) was developed by Zhang's group, based on the collateral effect of an RNA-guided and RNA-targeting CRISPR effector, Cas13a (Figure 4(a)) [57, 58]. The detection system is highly sensitive and specific because it is capable of single-molecule nucleic acid detection. Subsequently, they developed an enhanced SHERLOCK version 2 (SHERLOCKv2) detection system with a 3.5-fold improvement in detection sensitivity and lateral flow readout. SHERLOCKv2 has been used to detect dengue and Zika virus single-stranded RNA or mutations in clinical samples, showing great potential for multiplexable, portable, rapid detection of nucleic acids [59]. Recently, they combined RT-RPA technology with the SHERLOCK system (namely CRISPR diagnostics) to detect the S and ORF*1ab* genes of 2019-nCoV (Figure 4(b) and (c)) [60]. The CRISPR diagnostics-based test can be conducted in 1 hour and can be read using a dipstick. The analysis is performed at 37°C and 42°C, and its detection sensitivity is ten copies per microliter of input, exhibiting unique advantages, such as high sensitivity, specificity, speed, and suitability for point-of-care testing. However, this approach needs to be validated using real patient samples.

Unlike the CRISPR/Cas13a system, the CRISPR/Cas12a system is based on the collateral effect of Cas12a on single-stranded DNA (ssDNA). Chen and colleagues combined Cas12a ssDNase activation with RPA technology to create a new approach, named DNA endonuclease-targeted CRISPR trans reporter (DETECTR), with attomolar sensitivity for DNA detection (Figure 4(a)) [61]. DETECTR was also validated with clinical samples, showing the capacity for rapid, specific detection of human papillomavirus (HPV) [61]. Recently, DETECTR was investigated to identify the nucleic acid of 2019-nCoV. Lucia et al. applied the DETECTR (CRISPR-Cas12a and RT-RPA) to detect the RNA-dependent RNA polymerase (RdRp), ORF*1b*, and ORF*1ab* genes of 2019-nCoV using synthetic RNA fragments as samples [62]. Remarkably, all steps of the test were completed in 1 h, and results were visible to the unaided eye. The limit of detection for ORF*1ab* was ten copies/*μ*L. The advantages of this method are its portability and low cost (~US$2 per reaction). But this proposed approach also needs to be validated with clinical samples before commercialization. Another DETECTR-based 2019-nCoV detection strategy was developed by Chiu's lab [63]. They employed LAMP, CRISPR/Cas12, and lateral flow assay to detect the E and N genes of 2019-nCoV in clinical samples. This protocol supplied rapid (~30 min), low-cost, and accurate (100% specific vs. 90% specific for qRT-PCR) detection of 2019-nCoV in respiratory swab samples. Realistically, CRISPR/Cas-based 2019-nCoV detection technology is highly specific, rapid, and low cost, but the detection strategy also needs to be validated using clinical samples.

#### 2.1.5. Microfluidic-Based Detection

The abovementioned methods are based on relative quantification, because they require external calibration with genetic standards or inner reference DNA templates, resulting in unavoidable errors and other uncertainties. On the contrary, methods that do not need standard curves can provide a quantitative analysis of nucleic acids using absolute quantification of genetic copies. Recently, digital PCR and digital LAMP have been achieved with microelectromechanical and microfluidic technologies [64, 65].

Microfluidic or lab-on-a-chip techniques use microsized channels to process or manipulate fluids. Microfluidics has been widely utilized in various fields, including drug screening, tissue engineering, disease diagnostics, and nucleic acid detection [66, 67]. Based on its portability and ultralow sample consumption, microfluidics shows significant promises in clinical applications [68]. Regarding nucleic acid analyses, microfluidic devices aliquot diluted nucleic acid samples into hundreds to millions of discrete nanoliter chambers. The isolated chambers contain only one or zero target molecule according to a Poisson distribution. Consequently, the absolute copy number of target nucleic acid can be calculated from the number of positive and negative reactions, based on the Poisson distribution formulas [69, 70]. Both digital PCR and digital LAMP have employed microfluidics for pathogen analysis, which is suitable for COVID-19 detection. For instance, Ottesen and colleagues used digital PCR to amplify and analyze multiple genes on a microfluidic chip [71]. This chip consisted of parallel chambers and micromechanical valves. The micromechanical valves segmented chambers into independent PCR reactors after the sample flowed into chambers through connection channels. The chip was able to detect several kinds of genes with parallel sample panels. Digital PCR can also be conducted with droplets generated by the microfluidic chip. However, the detection of fluorescent signals in droplets requires special instruments, such as flow cytometers, which may limit its application in point-of-care testing. Additionally, the high temperature in PCR amplification tends to evaporate the reaction liquid (nanoliter or even femtoliter), leading to unacceptable errors. Using airtight devices or high pressure delays liquid evaporation but complicates the devices and increases testing costs.

**Figure 5:**
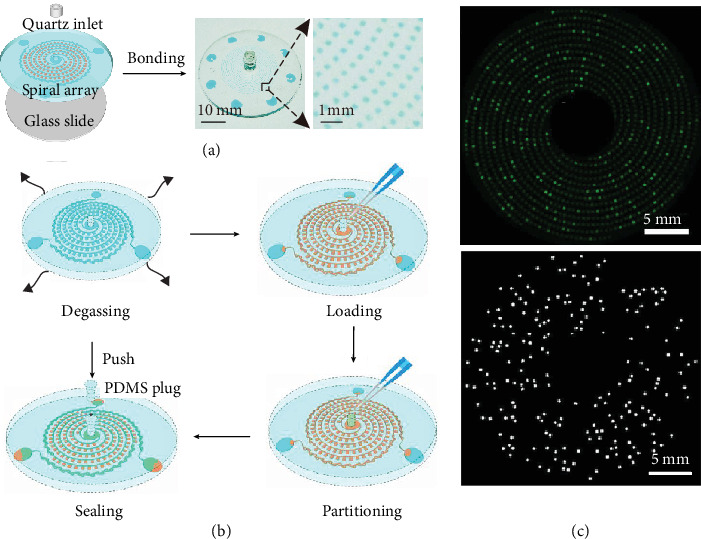
Microfluidic-based detection of 2019-nCoV. (a) Design and fabrication of a spiral chip. (b) Operation procedures for sample introduction and partition on the chip. PDMS: polydimethylsiloxane. (c) Raw (upper) and software-extracted images (down) of the chip after nucleic amplification. Adapted and copyright with permission (a–c) [70], American Chemical Society.

Digital LAMP is more compatible with microfluidics than digital PCR because it is executed at a moderate temperature. This simplifies microfluidic devices and reduces testing costs. Many microfluidic devices have been reported for nucleic acid detection using digital LAMP, such as self-digitization chips, self-priming compartmentalization chips, and droplet-generation chips [72–74]. As an example, Xia et al. designed a mathematical model using the Monte Carlo method according to the theories of Poisson statistics and chemometrics [70]. The mathematical model illustrated influential factors of the digital LAMP assay, guiding the design and analysis of digital LAMP devices. Based on the established mathematical model, they fabricated a spiral chip with 1200 chambers (9.6 nL) for pathogen detection (Figure 5(a)–(c)). This spiral chip operated at 65°C without visible liquid evaporation and achieved a quantitative analysis of nucleic acids over four orders of magnitude in concentration with a detection limit of 87 copies per mL. This portable gadget shows significant promise in future point-of-care testing.

Microfluidics, combined with enzyme-DNA nanostructures, is also applied to detecting 2019-nCoV. Ho et al. developed a modular detection platform (termed enVision) consisting of an integrated circuit of enzyme-DNA nanostructures for direct and versatile detection of pathogen nucleic acids from infected cells [75]. Built-in enzymatic cascades in the enVision microfluidic system supply a rapid color readout for detecting HPV. The assay is fast (<2 h), sensitive (limit of detection < 10 attmol), and readily quantified with smartphones. Recently, they adopted the enVision microfluidic system to detect 2019-nCoV [76]. Preliminary results showed that the enVision platform is sensitive, accurate, fast (within 0.5-1 h), and inexpensive (less than $1 per test kit). This novel platform works at room temperature and does not require a heater or special pumps, and it uses a minimal amount of samples, making it highly portable. However, this platform needs to be further validated with real clinical samples.

### 2.2. Target Antigen and Antibody

As mentioned above, the primary diagnostic methods are virological detection involving viral nucleic acids. Another approach to detection is with serological assays that measure antigens or antibodies present in the host. Such testing provides vital information about host exposure to 2019-nCoV and is useful for detection and surveillance purposes. For instance, this method greatly helps medical professionals to determine whether some recovered patients have a higher risk of reinfection. However, the disadvantage is that one should be cautioned that in the early stages of COVID-19 infection, the host's antibodies are often not within the detectable range of serological test kits. Besides, there was no proven evidence on the duration of IgM or IgG antibodies circulating in the host after recovery. It could be merely a short time frame for detection. As such, serological tests should not be solely used for COVID-19 diagnosis.

#### 2.2.1. Enzyme-Linked Immunosorbent Assay (ELISA)

Early diagnosis of 2019-nCoV infection is of utmost importance both for medical teams to manage patients effectively and for policymakers to curb the viral spread. Presently, ELISA in cell culture extracts has proven to be the working “gold standard” for laboratory diagnosis of 2019-nCoV [77]. ELISA is a plate-based assessment method for detecting and quantifying biomolecules, including peptides, proteins, antibodies, and hormones. ELISA techniques depend on specific antibodies to bind target antigens and a detection system to indicate the presence of antigen binding. In an ELISA, an antigen must be immobilized to a solid surface and then complexed with an antibody that is linked to an enzyme. Detection is accomplished by assessing the conjugated enzyme activity after incubation with a substrate to produce a measurable product [78]. Recently, coronavirus proteins have been widely used in ELISA to diagnose SARS-CoV or other viruses within the coronavirus family [79].

In a bold, novel approach, a team of infectious disease experts in Singapore utilized an ELISA against 2019-nCoV to ascertain that suspected subjects were infected with COVID-19 and discovered the connection between two COVID-19 clusters in the local community [80]. Using blood samples taken from alleged COVID-19 patients, the researchers detected antibodies targeting the spike protein that prevented the virus from killing cells in laboratory tests. They verified that a couple allegedly infected with COVID-19 had the disease because they had exceedingly elevated levels of virus-specific antibodies in their blood. Interestingly, PCR tests on the couple yielded negative results. Because the couple had recovered from the 2019-nCoV infection, they had no viral genetic materials in their bodies, but the antibodies persisted. There were also other reports of using ELISA to diagnose 2019-nCoV infection [81, 82]. Each study confirmed the high reproducibility and specificity of ELISA in diagnosing COVID-19 patients accurately in clinics.

#### 2.2.2. IgG/IgM Lateral Flow Assay (LFA)

Research has established that the presence of immunoglobulin M (IgM) indicates a primary defense against viral infections. This IgM defense occurs before the production of high-affinity and adaptive immunoglobulin G (IgG) that is critical for prolonged immunity and immunological memory [83]. From a previous study on SARS infections, both IgM and IgG antibodies could be detected in the patient blood after 3-6 days and beyond 8 days, respectively [84]. Given that 2019-nCoV belongs to the same family of coronaviruses including MERS and SARS, 2019-nCoV should also generate IgM and IgG antibodies in infected humans. Therefore, the detection of IgM and IgG antibodies may provide epidemiologists with crucial information on viral infection of test subjects, allowing them to adjust policies to combat the pandemic more effectively.

**Figure 6:**
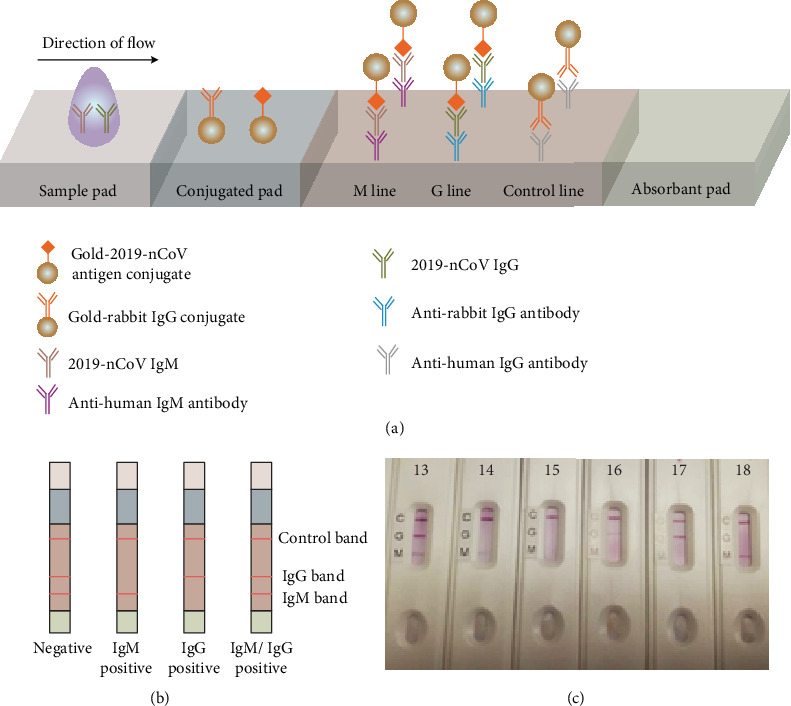
Lateral flow assays for detecting 2019-nCoV antigens or antibodies to 2019-nCoV. (a) Diagram showing LFA-based quick detection of 2019-nCoV in an IgM-IgG combined antibody assay. (b) A diagram showing different test results. (c) Representative assay test showing results from blood of different patients. Patient #13: IgM and IgG positive; #14: IgM weak positive; #15: IgM and IgG negative; #16: IgG weakly positive; #17: IgG positive only; #18: IgM positive only. Adapted and copyright with permission (c) [84], Wiley.

Point-of-care lateral flow immunoassays are performed qualitatively to quickly determine the presence of 2019-nCoV by detecting anti-2019-nCoV IgM and anti-2019-nCoV IgG antibodies in human plasma, serum, or whole blood. A typical device is shown in Figure 6(a). Reddish-purple lines in the readout indicate the presence of 2019-nCoV IgM and IgG antibodies in the sample. LFA is based on the lateral chromatographic flow of reagents that bind and interact with the sample. As the sample flows through the test device, starting at the sample pad region, the anti-2019-nCoV IgM and IgG antibodies, if present, bind tightly to 2019-nCoV antigen-labeled gold nanoparticles, located on the conjugated pad. When conjugated products in the sample continue to move up the strip, anti-2019-nCoV IgM antibodies and anti-2019-nCoV IgG antibodies bind to anti-human IgM (M line) or anti-human IgG (G line), respectively. No visible lines can be seen when the specimen does not contain anti-2019-nCoV antibodies because no labeled complexes bind at the test zone. IgG-labeled gold colorimetric nanoparticles serve as the control when they bind to anti-rabbit IgG antibodies at the control line (C) (Figure 6(b)). LFA has proven useful in detecting 2019-nCoVIgM/IgG antibodies in clinical studies, demonstrating 88.66% test sensitivity and 90.63% specificity in human blood, serum, and plasma samples. Results from six patients are shown in Figure 6(c). Common 2019-nCoV detection methods are summarized in Table 1.

### 2.3. Supplementary Detection Methods

Various diagnostic techniques have been used to complement RT-PCR and antibody-antigen serological testing. These include chest computed tomography (CT) and transmission electron microscopy (TEM). Each has its place in diagnostic settings and can serve as a complementary diagnostic tool to aid medical investigators in diagnosing 2019-nCoV accurately in suspected COVID-19 patients.

#### 2.3.1. Chest Computed Tomography (CT)

**Figure 7:**
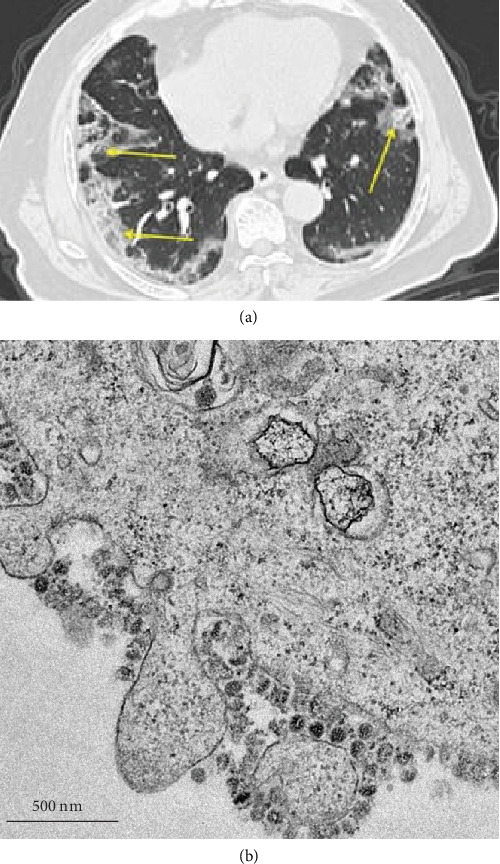
Computed tomography and transmission electron microscopy characterizations of 2019-nCoV. (a) Cross-sectional noncontrast enhanced chest CT radiographs of a man's lungs with COVID-19. The figure shows enlarged lesions and increased density of the lesions at the outer edge of the lungs (yellow arrows) [93]. (b) A TEM image of the 2019-nCoV grown in cells at the University of Hong Kong [97].

In clinics, medical imaging tools are indispensable and form an essential component of viral diagnosis, as well as for monitoring viral progression [85]. They have also been used for follow-up in outpatient settings for coronavirus-related pulmonary disorders. Just like both SARS and MERS, pulmonary complications in COVID-19 patients have been observed. Learning from the well-documented SARS and MERS studies, CT imaging results in the acute and chronic periods of COVID-19 are invariant, but not always present [86–89]. Evidence is found in previous studies on SARS and MERS. The glass opacities observed are not always found in COVID-19 patients. Crucially, preliminary imaging discoveries indicate that COVID-19 yields nonspecific results as well [90–92]. Radiologists are presently striving to any characteristics specific to COVID-19, although present medical information remains limited. Given the precarious situation, there is a pressing need for alternative, complementary diagnostics. CT is one example. COVID-19 patients often develop “ground glass” lung opacities [93]. As such, a CT imaging scan can readily identify lung abnormalities in human subjects, thereby enabling early treatment against COVID-19. CT has demonstrated some common imaging characteristics in COVID-19 patients. These features include bilateral, multifocal, ground glass opacities, with a peripheral distribution (Figure 7(a)) [93]. Crucially, more than half of 90 patients under study presented multilobar involvement and lesions more prominently in the lower lobes of their lungs. Given its feasibility and ease of use, CT has become an essential tool for the 2019-nCoV infection diagnosis. From a radiological perspective, the advantages of using CT imaging could expedite the rate of diagnosis. It also supports the current shortage and heavily reliant on technical know-how during RT-PCR testings. Nonetheless, one limitation is that it should be cautiously utilized as a diagnostic approach because there are no proven, evidence-based clinical benefits of using CT. It could also cause false securities if results are negative. Other limitations include requirements of relatively high-dose CT scans and long-term, continuous usage, which can altogether be logistically challenging and deplete additional medical resources.

#### 2.3.2. Transmission Electron Microscopy (TEM)

Transmission electron microscopy (TEM) has been used for many years and has had a profound impact on our understanding of illnesses, including viral infections. The thousandfold enhanced resolution provided by TEM enables investigators to visualize viral morphology and to classify viruses into families [94].

Mechanistically, TEM operates based on interactions between electrons emitted from a source and materials under examination. In the present context, it is usually 2019-nCoV in a cellular sample [95]. The detector collects a multitude of signals from transmitted electrons, before processing them to reveal viral morphology and location within cells [96]. Typical specimen preparation for TEM includes sample fixation, embedding, sectioning, staining, and loading onto the TEM copper grids [94, 97, 98]. 2019-nCoV sampling typically uses supernatants from patient airway epithelial cells. Infected cells are fixed and dehydrated before embedding in resin. A negatively stained, film-coated grid for examination is similarly prepared for contrast enhancement. 2019-nCoV virus particles seen with TEM are shown [97] (Figure 7(b)). TEM enables microbiologists to rapidly diagnose patients with a single examination of a single tissue sample.

## 3. Medical Treatment

There are three general approaches to develop potential antiviral treatments of the human coronavirus. Firstly, standard assays may be used to evaluate existing broad-spectrum antiviral drugs. Secondly, chemical libraries containing existing compounds or databases may be screened. Thirdly, specific, new medications based on the genome and biophysical understanding of 2019-nCoV can be designed and optimized. Therefore, this section will discuss some of the potential 2019-nCoV therapeutics obtained through these general approaches. Besides chemical and biologic drugs commonly used in antiviral therapies, we further elaborate on how nanomaterials, nutritional interventions, traditional Chinese medicine, and stem cell therapy can be potentially used for treatment or as an adjuvant to reduce the mortality and morbidity rate of 2019-nCoV patients. Finally, to end this section, we highlight vaccines as a key therapeutic option to eradicate COVID-19 through herd immunity without getting the disease.

### 3.1. Chemical Drugs

There are currently no approved antiviral drugs to treat COVID-19, and patients must depend upon their immune systems to combat the infection. A full-fledged treatment plan has yet to emerge, and both academia and pharmaceutical companies are racing to develop new treatments and vaccines to address COVID-19. Research into the cellular and molecular pathogenesis of 2019-nCoV has provided essential insights with the hope of developing viable therapies. While researchers are working on cures or preventive measures for COVID-19 [99], a more robust, efficient, and economical way to tackle the disease is to repurpose existing drugs into a viable therapeutic strategy. Drug repurposing, also termed drug repositioning, refers to the process of discovering new therapeutic applications for existing drugs. It offers various advantages over traditional de novo drug discovery, i.e., reduced cost and drug development time, established drug characteristics, and, most importantly, established safe dosages for human use [100]. Repurposed drugs often negate the need for phase 1 clinical trials and can be used immediately [100, 101]. At present, repurposed drugs are the only option available at treatment centers for COVID-19 patients. As COVID-19 is a viral infection, the most obvious choices for repurposed drugs come from known antiviral drugs [102]. Antiviral creation strategies focus on two approaches: targeting viruses or targeting host cell factors. In this section, we will review antiviral drugs prescribed or proposed against COVID-19 based on the antiviral drug creation strategies.

#### 3.1.1. Entry Inhibitors

**Figure 8:**
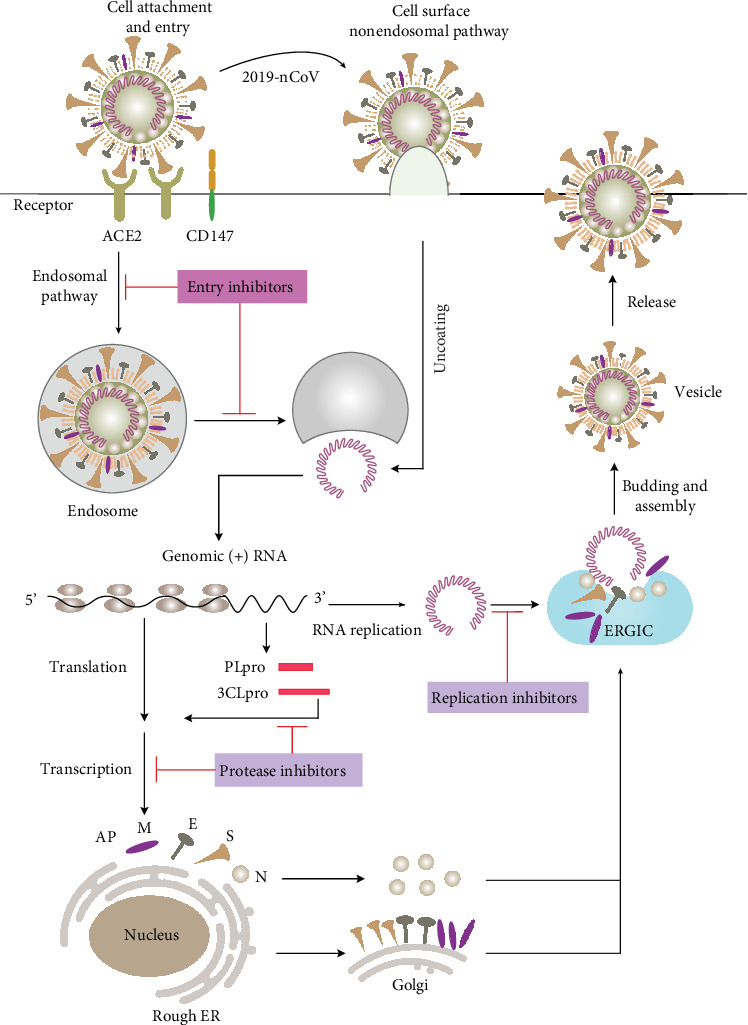
Proposed schematic of 2019-nCoV replication and potential treatment options targeting the coronavirus replication cycle. ACE2: angiotensin-converting enzyme 2; +: positive-strand RNA; AP: accessory protein; E: envelope protein; ER: endoplasmic reticulum; N: nucleocapsid protein; M: membrane protein; ERGIC: endoplasmic reticulum-Golgi intermediate compartment; S: spike glycoprotein.

**Figure 9:**
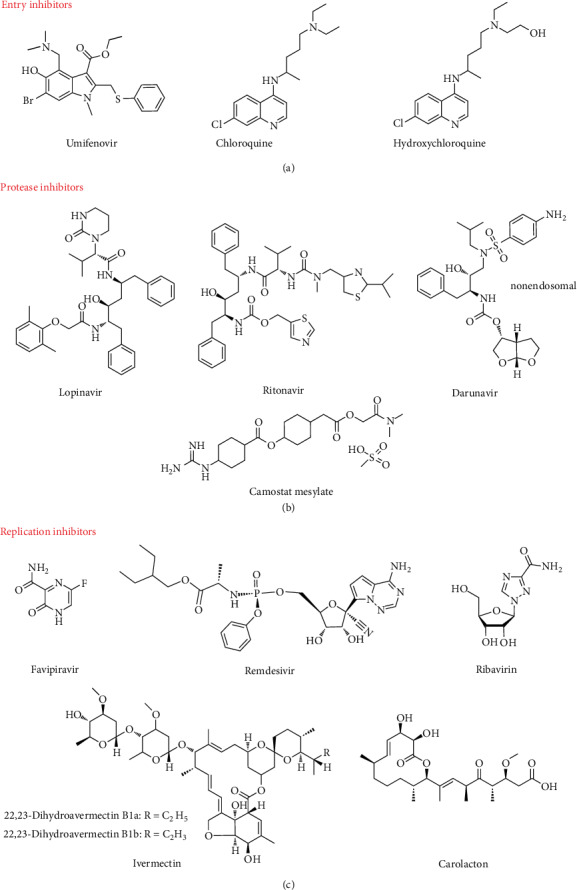
Chemical structure of drugs used to treat COVID-19. (a) Chemical structures of entry inhibitors. (b) Chemical structures of protease inhibitors. (c) Chemical structures of replication inhibitors.

When an infection occurs, the virus gains entry into a host cell by attaching itself to the host cell surface (Figure 8) [103]. This relies on numerous interactions between the virion surface and the specific proteins on the cell membrane. In general, these surface proteins have other functions but are serendipitously recognized by the virus as entry receptors [103]. Molecules that prevent such recognition, either by competitive binding or by downregulating the receptors, are known as viral entry inhibitors (Figure 9(a)). These inhibitors are valuable as therapeutics since blocking infection early in the life cycle reduces cellular and tissue damage associated with viral replication and production of viral progeny.

As mentioned above, coronavirus particles comprise four structural proteins: the S, E, M, and N proteins [104–107]. The S protein is the most crucial in viral attachment, fusion, and entry [108]. It comprises two subunits. S1 facilitates attachment to the host cell receptor, while S2 mediates membrane fusion of the virion and the host cell. As mentioned above, viruses have specific attachment sites. SARS-CoV recognizes ACE2 as its host receptor, while MERS-CoV recognizes dipeptidyl peptidase 4 [109, 110]. Like SARS-CoV, 2019-nCoV also targets host ACE2 [111–113]. Biophysical and structural analysis indicates that the 2019-nCoV S protein binds ACE2 with higher affinity than the SARS-CoV S protein [16]. Therefore, it is vital to target ACE2 for the development of viral entry inhibitors.

To the best of our knowledge, not much is known about ACE2-specific inhibitors that are commercially available or under commercial development [114]. However, ACE2 stimulators have been used in the treatment of hypertension, cardiac diseases, and diabetes mellitus to regulate the renin-angiotensin system [115, 116]. There are also ACE inhibitors known for treating the diseases mentioned, but these lack inhibitory activity toward ACE2 due to their distinct substrate-binding pockets [116–119]. In brief, there are concerns that both ACE2 stimulators and ACE inhibitors can increase the expression of ACE2, which in turn may increase susceptibility to viral host cell entry [120, 121]. Much work needs to be done on ACE2-targeting drugs, and controversial issues that lie beyond the treatment pathway need to be addressed soon.

A small antiviral molecule, umifenovir, has entry inhibitory effects on the influenza virus. Umifenovir targets hemagglutinin for its anti-influenza virus effect [122–124]. Hemagglutinin, a viral cell surface protein, facilitates infection by undergoing a conformational change when the virus binds to host cells [122]. Umifenovir interacts with hemagglutinin to stabilize it against low pH-induced conformational change via the formation of an extensive network of noncovalent interactions that prevent hemagglutinin-mediated membrane fusion [122, 124]. It also interacts with phospholipids by altering membrane fluidity [125], which is vital for the fusion process. This is most likely due to umifenovir's molecular interactions (bearing both the H donor and acceptor groups) with the interfacial region of the lipid bilayer by competing for the hydrogen bonding of phospholipid C=O groups with water molecules [126]. This renders lipid bilayers of host cells less prone to viral fusion [125]. No studies have shown that umifenovir is effective in inhibiting SARS-CoV or 2019-nCoV. Wang et al. reported that 4 patients with mild/severe COVID-19 recovered after prescription of combined lopinavir/ritonavir, arbidol (umifenovir), and Shufeng Jiedu Capsule (a traditional Chinese medicine) [127]. On the other hand, Dong et al. found in an *in vitro* study that arbidol may effectively inhibit 2019-nCoV infection at a concentration of 10-30 *μ*M [128].

Chloroquine, also a small molecule, is a quinine analog used to prevent and treat malaria. Similar to umifenovir, chloroquine exhibits its inhibitory effect on influenza by pH stabilization. Chloroquine is a weak base and becomes protonated intracellularly in a manner described by the Henderson-Hasselbalch law [129]. It can raise lysosomal pH to facilitate autophagy intracellularly [130–132]. Chloroquine also alters the signaling pathway of enzymes, causing enzyme glycosylation, ultimately inhibiting viral replication in host cells [133, 134]. Liu et al. claimed that chloroquine could inhibit SARS-CoV entry by changing glycosylation of the ACE2 receptor and S protein [135]. Chloroquine's effective inhibition of SARS-CoV was demonstrated *in vitro* on primate cells and human rectal cells [136, 137]. Hydroxychloroquine is a derivative of chloroquine with an additional hydroxyl group. These two chloroquines share similar structures and mechanisms. Both have shown *in vitro* antiviral activities toward 2019-nCoV [138–140]. Hydroxychloroquine was more effective than chloroquine in inhibiting 2019-nCoV *in vitro* on primate cells [141]. Until now, chloroquine has shown apparent efficiency and safety against 2019-nCoV in clinical trials conducted in China [139]. Currently, chloroquine or hydroxychloroquine has been administered to hospitalized 2019-nCoV patients on an uncontrolled basis in various countries, including China and the USA [42]. However, it must be noted that chloroquine and hydroxychloroquine cause ocular toxicity [142]. Hydroxychloroquine is reportedly less toxic than chloroquine, making it more attractive as a prescription drug [135, 143]. Nevertheless, more investigation and clinical trials are needed to evaluate further their efficacy and safety in treating 2019-nCoV.

#### 3.1.2. Protease Inhibitors

Proteases are essential enzymes that regulate cell life processes such as cell growth and death, blood clotting, inflammation, fertilization, and infection [103]. Viral entry into host cells requires S protein priming by host proteases, which subsequently enables the fusion of viral and cellular membranes [113]. Membrane fusion enables the release of the viral genome into the host cytoplasm, initiating RNA translation into protein. Most viruses also encode their proteases to protect viral proteins by modulating host cell responses. While proteases are vital for cell life processes, they have become promising targets for antiviral therapeutic agents. Protease inhibitors prevent viral replication by binding selectively to viral proteases or blocking proteolytic cleavage of protein precursors necessary for the production of infectious particles [144]. It is noteworthy that protease inhibitors were a major therapeutic breakthrough of antiviral drug design in the mid-1990s for the treatment of HIV. Most HIV protease inhibitors have found prominent clinical use (Figure 9(b)).

Coronavirus S proteins can be primed by a multitude of proteases [145]. Hoffmann et al. demonstrated that the S protein of 2019-nCoV could be primed by serine protease TMRPSS2 [113]. Similarly, both SARS-CoV and MERS-CoV can be activated by other TMPRSS family members [145]. TMPRSS family proteases are widely expressed in the respiratory tract [145, 146], which is likely the reason that coronaviruses cause acute respiratory distress syndrome. Upon successful priming, the viral genome encoding RNA and several nonstructural proteins, including coronavirus main protease (3CLpro), papain-like protease (PLpro), and RdRp, are released [147–149]. The single-stranded positive RNA is translated into viral polyproteins by ribosomes in the host cell cytoplasm. The polyproteins are then cleaved into effector proteins by viral proteases: 3CLpro and PLpro. PLpro also acts as a deubiquitinase that may remove specific host cell proteins (e.g., interferon regulatory factor 3 (IRF3) and nuclear factor kappa-light-chain-enhancer of activated B cells (NF-*κ*B)), thus weakening the immune system [147, 149, 150]. Both host and viral proteases are essential therapeutic targets in the case of COVID-19.

Camostat mesylate is a small molecule that has shown an excellent therapeutic effect for chronic pancreatitis treatment by targeting proteases [113, 151, 152]. Camostat mesylate primarily inhibits enzymatic autodigestion of the pancreas [153]. *In vivo* studies on rats with pancreatic fibrosis showed that camostat mesylate inhibits inflammation, cytokine expression, and fibrosis in the pancreas [154]. It has an additional clinical benefit for pancreatic pain by preventing enzyme-evoked activation of pain receptors [155]. As mentioned above, the TMPRSS family, especially TMRPSS2, is most likely the protease targeted by a coronavirus. Camostat mesylate inhibits TMPRSS2 activity on primate cells *in vitro*, completely blocking membrane fusion between the host cell and the viral MERS-CoV particle [156]. Zhou et al. claimed that camostat mesylate displays an inhibitory effect in mice for SARS-CoV infection [152]. Recent research by Hoffmann et al. showed a promising *in vitro* inhibitory effect of this serine protease inhibitor in SARS-CoV and 2019-nCoV on human lung cells, showing potential as a viable option for COVID-19 treatment [113]. Unfortunately, *in vitro* and *in vivo* data for camostat mesylate against coronaviruses are limited. More investigation is required to evaluate camostat mesylate as a potential therapeutic against COVID-19.

Lopinavir-ritonavir is a coformulated antiretroviral drug with excellent efficacy against HIV-1. The lopinavir has a core molecular structure identical to ritonavir. The 5-thiazolyl end group and 2-isopropylthiazolyl group in ritonavir are replaced by the phenoxyacetyl group and a modified valine, respectively, in which the amino terminus has six-membered cyclic urea attached. In brief, lopinavir is a potent protease inhibitor developed from ritonavir with high specificity for HIV-1 protease [103]. It represents a higher proportion of the coformulation. Lopinavir contains a hydroxyethylene scaffold mimicking a standard peptide bond cleavable by HIV-1 protease [157]. This results in the production of noncontagious viral particles. On the other hand, ritonavir binds to HIV-1 protease, interrupting the maturation and production of viral particles [158]. A clinical study from Hong Kong has shown that the combination of lopinavir-ritonavir and ribavirin treatment for 152 patients against SARS-CoV had an overall favourable clinical response [159]. It has been demonstrated that lopinavir-ritonavir targets 3CLpro of 2019-nCoV and further indicated that 3CLpro might also be the targets of protease inhibitors for other coronaviruses [160]. Regrettably, a recent clinical trial using lopinavir-ritonavir in Wuhan, China, reported that 199 hospitalized adult patients infected with 2019-nCoV did not benefit from the treatment [161]. Given such conflicting clinical data, physicians must carefully weigh lopinavir-ritonavir as a COVID-19 treatment.

Darunavir is another antiretroviral protease inhibitor drug effective against HIV-1. Darunavir is designed for multidrug-resistant HIV-1 protease variants, due to its molecular structure, which introduces more hydrogen bonds compared to conventional antiretroviral medicines. In general, changes in van der Waals and hydrogen bonding interactions between inhibitors and proteases affect the potency of antiretroviral drugs [162]. Aside from enzymatic inhibition, darunavir inhibits protease dimerization [163]. The dimerization of HIV protease is essential for the acquisition of its proteolytic activity for the maturation of viral particles [163]. Lin et al. claimed that darunavir inhibits 2019-nCoV. The group has used molecular modeling to evaluate darunavir binding to 3CLpro and PLpro proteases and found targeted activity against the latter [164]. Nevertheless, the therapeutic effect of darunavir in COVID-19 clinical cases remains untested [164]. This may be in part due to potential side effects, such as liver damage and severe skin rashes [103, 165]. These contraindications must be carefully evaluated if darunavir is to be considered a potential therapeutic agent for COVID-19.

#### 3.1.3. Replication Inhibitors

Polymerases are enzymes essential for viral replication to produce viral progeny. Viral DNA and RNA polymerases are responsible for duplicating the viral genome and facilitating transcription and replication [103]. Replication inhibitors (Figure 9(c)) interfere with the production of viral particles by blocking enzymatic activity, ultimately causing chain termination during viral DNA or RNA replication [166]. There are four types of viral polymerases in viruses: RNA-dependent RNA polymerases, RdRp, DNA-dependent RNA polymerases, and DNA-dependent DNA polymerases.

In the section Protease Inhibitors, we mentioned that RdRp is released upon successful priming. RdRp is a necessary polymerase that catalyzes the replication of RNA from an RNA template for coronaviruses [167]. Release of RdRp from the virus initiates the synthesis of a full negative-strand RNA template to be used by RdRp to replicate more viral genomic RNA, which eventually turns host cells into virus factories [147]. Therefore, RdRp is an attractive therapeutic target to prevent host cells from producing viruses.

Ribavirin is a synthetic guanosine nucleoside analog that mimics purines, including inosine and adenosine, and ribavirin has been used in the treatment of respiratory syncytial virus [168]. It has only one ring at the heterocyclic base, compared with guanine's two rings. Notably, ribavirin has a ribose sugar moiety with a hydroxyl group at the 2′-carbon position, enabling preferential activity in RNA-related metabolism [168, 169]. Ribavirin inhibits cellular enzyme and inosine monophosphate dehydrogenase involved in purine nucleotide biosynthesis [170, 171]. Ribavirin is also known for its inhibitory effect on viruses by forcing viral genome replication to become catastrophically error-prone. It is likely that as a nucleoside analog, ribavirin is incorporated by RdRp into the newly synthesized viral genome, where it induces mutagenesis [170, 172]. Although ribavirin has proven effective against viral infections, its mechanism of action has not been firmly established, and there are several proposed mechanisms of action that require further validation [168, 173]. Ribavirin was initially used in treating SARS; however, ribavirin treatment lacked an *in vitro* antiviral effect and caused adverse side effects including anemia, hypoxemia, and decreased hemoglobin levels [174]. However, ribavirin was used as the primary treatment during the MERS outbreak [175]. In general, clinical studies of ribavirin treatment for SARS and MERS did not show strong evidence of efficacy against these coronaviruses [176–178]. There have been no studies of ribavirin's efficacy against COVID-19. Therefore, the use of ribavirin remains controversial and requires more investigation for a better understanding of its mechanism of action, efficacy, and toxicity, even though it is a widely available drug.

Favipiravir is a synthetic guanine analog frequently used for influenza treatment [179]. Structurally, favipiravir is closely related to ribavirin, in which it shares the same carboxamide moiety [180]. While ribavirin interacts with the viral polymerase directly, favipiravir must be phosphoribosylated by cellular enzymes to its active form, favipiravir-ribofuranosyl-5′-triphosphate (RTP) [181, 182]. The viral polymerase mistakenly recognizes favipiravir-RTP for a purine nucleotide, thereby disrupting viral genome replication [181, 182]. Favipiravir has not been used against SARS and MERS previously, but interestingly, it has been shown to reduce viral infection of 2019-nCoV [138, 183]. In a clinical study involving 80 patients infected with 2019-nCoV, conducted in Shenzhen, China, favipiravir showed better efficacy than lopinavir-ritonavir in terms of disease progression and viral clearance [183]. Another clinical study involving 240 patients with COVID-19 conducted in Hubei Province, China, also demonstrated that those treated with favipiravir had a higher recovery rate compared to those treated with umifenovir (preprint) [184]. More clinical data are needed to validate favipiravir's efficacy and safety in 2019-nCoV treatment.

Remdesivir is a trial synthetic adenosine analog that has not yet been clinically approved [185]. It was synthesized and developed by Gilead Science in 2017 for Ebola virus infection [186]. Remdesivir needs to be metabolized into its active form, GS-441524, to initiate its activity. The active form of remdesivir inhibits viral RNA polymerase and evades proofreading by viral exonuclease, causing an interruption in viral RNA production [138, 185, 186]. It has been demonstrated that remdesivir is effective against MERS-CoV infection *in vivo* and 2019-nCoV *in vitro* [138, 187], showing great potential as a therapeutic agent for 2019-nCoV. The drug is currently being validated in clinical trials [188]. Given that antiviral drugs have previously demonstrated reasonable inhibition of coronaviruses and therapeutic efficacy against coronavirus outbreaks, umifenovir, chloroquine, hydroxychloroquine, lopinavir-ritonavir, and ribavirin have been recommended in the latest guidelines for diagnosis and treatment of COVID-19, updated on 17 February 2020 [189].

Recent studies also demonstrated that some antibiotics potentially inhibit 2019-nCoV replication. Anderson et al. (preprint) recently developed the first bat genome-wide RNA interference (RNAi) and CRISPR libraries and identified MTHFDI as the critical host factor for viral infections [190]. MTHFDI is a trifunctional enzyme involved in the one-carbon (C1) metabolic pathway, participating in the cellular production of purine, dTMP, and methyl groups [191]. Anderson et al. demonstrated that purine synthesis activity of MTHFDI is an essential activity for viral replication, making MTHFDI a potential target for developing antiviral drugs [190]. They further explained that an MTHFD1 inhibitor, carolacton, restricts replication of influenza virus, mumps virus, Melaka virus, Zika virus, and, most importantly, 2019-nCoV [190]. Carolacton is a secondary metabolite derived from the mycobacterium *Sorangium cellulosum*. It is a macrolide ketocarbonic acid. Carolacton has been studied as an antibacterial compound against biofilms of pathogenic *Streptococcus mutans* and growth of pathogen *Streptococcus pneumoniae* [192, 193]. It has no toxic effect against eukaryotic cells [194]. It has recently been identified as a potent inhibitor of MTHFDI, and its mechanism of action is presumably due to the ability of carolacton to bind with MTHFDI [194]. More research is needed to validate the mechanism of action, efficacy, and safety of carolacton as a possible treatment for COVID-19. On the other hand, ivermectin is originally a medication used to treat parasite infestation. It comprises different analogs of avermectin: 22,23-dihydroavermectin B1a and 22,23-dihydroavermectin B1b, at a ratio of 4 : 1 [195]. They are macrolide antibiotics isolated from the fungus *Streptomyces avermitilis*. It has reportedly stopped HIV-I proliferation by inhibiting interaction of the retroviral integrase protein with adapter protein (importin), responsible for the nuclear protein import cycle [196]. Caly et al. reported that ivermectin successfully inhibited 2019-nCoV *in vitro* but the mechanism of action is unclear [197]. Since ivermectin is an approved drug, it shows great potential as a therapeutic agent for COVID-19. *In vivo* work or clinical trials need to be done to confirm its efficacy and safety for treatment against COVID-19. Potential drugs for COVID-19 are summarized in Table 2.

### 3.2. Nanodrug Delivery System

Nanomaterials have recently been utilized for the treatment of diseases such as cancer [198–200] and various types of infections [201, 202]. The ease of modification of surface properties, large surface area [203], and multivalent interactions with targets [204] imbues nanomaterials with massive potential as highly efficacious COVID-19 therapeutic options. However, to the best of our knowledge, no nanoparticle treatment option has been applied to COVID-19. Nonetheless, results obtained from nanoparticle research against other viruses have shown promising potential. For example, Fujimori et al. utilized a CuI nanoparticle to treat H1N1 influenza through the generation of reactive oxygen species (ROS) that inactivate the virus [205]. Silver nanoparticles also show much promise in treating COVID-19 with their broad antiviral properties against a multitude of viruses, including HIV, hepatitis B virus, herpes simplex virus, respiratory syncytial virus, and monkeypox virus [206]. The broad antiviral properties of silver nanoparticles and the generality of ROS inactivation suggest that these nanoparticles can be utilized therapeutically without any modifications. Nanoparticles could also be used for drug delivery. Recently, Herold and Sander demonstrated the use of pulmonary surfactant-biomimetic nanoparticles to encapsulate a stimulator of interferon gene (STING) agonist, 2′,3′-cyclic guanosine monophosphate-adenosine monophosphate, as an adjuvant in a variety of influenza vaccines [207]. Using nanoparticles as a delivery agent, immune cells were activated without excessive inflammation in the lung. This could provide a considerable benefit for use in COVID-19 vaccines in the future, but as the field is still relatively new, especially in medicinal applications, safety should remain a key consideration in the adoption of nanoparticles in humans.

### 3.3. Biologic Drugs

In addition to chemical medicines, another vital form of therapy for COVID-19 may be the use of biologics. Currently, interferon-*α*2b nebulization of 100,000 to 400,000 IU/kg twice a day for 5 to 7 days is one of the main treatments for COVID-19 in children, and it has demonstrated efficacy in reducing the viral load during early stages of infection [208, 209]. Another promising biologic drug is convalescent plasma, the plasma of patients who have recovered from COVID-19 [210, 211]. Antibodies in the donated plasma could confer temporary, passive immunity against COVID-19, allowing patients time to develop active immunity. Clinical trials are currently ongoing [212, 213], and preliminary results announced from the Chinese hospitals have been promising.

On the other hand, human monoclonal antibodies or their fragments developed in the lab have shown encouraging results as well. Tian et al. confirmed the binding of a human monoclonal antibody CR3022 to the receptor-binding domain (RBD) of 2019-nCoV with high affinity [214], highlighting the therapeutic potential of CR3022 toward COVID-19, though further *in vitro* and *in vivo* studies are required before it could be used clinically.

### 3.4. Nutritional Interventions

Another supportive treatment for COVID-19 involves dietary interventions. Various research studies have shown supplementation of multiple vitamins and minerals such as vitamins A, C, and D and zinc can reduce the severity of respiratory infections [215–221]. However, most of these studies targeted children below the age of 5 who were suffering from malnourishment or preexisting diseases. Therefore, vitamin and mineral supplementation may offer more significant benefits to COVID-19 patients in developing countries. Moreover, aggressive supplementation of calories and protein in nutritionally at-risk patients has shown significant benefits in reducing mortality [222]. Using a modified Nutrition Risk in Critically Ill (mNUTRIC) score, Kalaiselvan and coworkers demonstrated that 42.5% of mechanically ventilated patients have high nutritional risks (mNUTRIC score ≥ 5), accompanied by long intensive care unit (ICU) stays and high mortality rates [223].

An estimated 5% of COVID-19 patients require ICU care, and of these critically ill patients, most need mechanical ventilation [224, 225]. Therefore, nutritional intervention using aggressive calorie and protein supplementation may provide substantial benefits to a significant number of critically ill patients. Evidence of such benefits may be provided by the clinical trial (NCT04274322) that is expected to end in July 2020.

### 3.5. Traditional Chinese Medicine

Traditional Chinese medicine (TCM) is considered a prospective supplementary treatment of COVID-19, due to its impressive performance in treating SARS in 2003 [226]. First, TCM shows a generalized antiviral effect through direct inhibition of viruses and control of inflammation. For example, Weng et al. reported that the *Smabucus Formosana Nakai* (a traditional medicinal herb) ethanol stem extract displayed strong anti-HCoV-NL63 activity [227]. Moreover, TCM can alleviate damage induced by inflammatory reactions and immune responses initiated by viral infections. Single and combined Chinese medicines could mitigate the cytokine storm by clearing the heat and toxicity in the body. For instance, TCM approaches were adopted to prevent and treat SARS in 2003 and H1N1 influenza in 2009 [228]. As of February 17, 2020, over 85.2% of total confirmed cases (over 60,000 cases) had been treated with TCM, showing that TCM yields excellent outcomes. Notably, in a trial of 102 cases with mild symptoms, TCM achieved remarkable therapeutic effects, demonstrated by faster clinical symptom disappearance and reduction of fever, shorter disease course, higher cure rate (by 33%), and lower rate of moderate-to-severe cases [229]. To date, the National Health Commission (NHC) of the People's Republic of China has published seven editions of the guidelines for diagnosis and treatment of COVID-19 [25]. Since the fourth version, a list of TCM prescriptions (including TCM soup and TCM capsules) has been recommended for patients based on the stage of the disease and their symptoms [230].

According to the 7^th^ edition of the guidelines, there are three kinds of TCM prescriptions recommended for different stages of patients: the medical observation period, the clinical treatment period, and critical condition (details in Table 3) [25]. Among TCM recipes, the Qing Fei Pai Du Decoction is strongly recommended for treatment of COVID-19 by the NHC of the People's Republic of China, because it gave a cure rate of over 90% of COVID-19 patients in a clinical trial involving 701 confirmed cases [231]. Another TCM recipe, Xue Bi Jing Injection is specifically recommended for treating critically ill COVID-19 patients, because it suppresses severe sepsis, according to the China Food and Drug Administration. It also promoted significant improvement in cases of severe community-acquired pneumonia (CAP) [232]. Therefore, TCM could be an alternative prophylactic approach to COVID-19 and a supplementary treatment in combination with western medicine to cure COVID-19.

### 3.6. Stem Cell Therapy

Stem cell therapy is a promising treatment strategy for degenerative diseases, including Huntington's disease, Parkinson's and Alzheimer's diseases, and chronic diseases such as cardiac failure and diabetes [233]. A clinical study showed that transplantation of mesenchymal stem cells (MSCs) significantly lowered the mortality of patients with H7N9-induced acute respiratory distress syndrome (ARDS), with no harmful effects [234]. As H7N9 and 2019-nCoV share similar genome structures and corresponding infection mechanisms, as well as related clinical symptoms (lung failure), MSC-based therapy could be a possible alternative for treating COVID-19. Currently, stem cell-based therapy for COVID-19 is being conducted by different hospitals in China. Doctors from Baoshan Hospital (Yunnan province, China) used human umbilical cord mesenchymal stem cells (hUCMSCs) to treat a 65-year-old critically ill woman with COVID-19. Two days after the 3rd injections of stem cells, the woman recovered, and the throat swab test for COVID-19 turned negative [235]. Another clinical trial involving stem cell therapy was conducted in seven confirmed COVID-19 patients in different clinical stages in Beijing Youan Hospital (Beijing, China). Two to four days after intravenous transplantation of CE2^−^ MSCs, all symptoms such as high fever, weakness, and shortness of breath disappeared in all seven patients without observed adverse effects, indicating that MSCs can cure or significantly improve functional outcomes [236]. There are at least 12 other trials using stem cells to treat COVID-19 in China, according to the WHO report.

### 3.7. Other Treatments

Vaccines are another promising treatment to prevent or cure specific viruses. Currently, there is no effective vaccine against 2019-nCoV. Fortunately, two COVID-19 vaccines are undergoing clinical trials. The first is Moderna's mRNA-1273, an mRNA vaccine, which started at KPWHRI in Seattle, USA, on March 16, 2020 [237]. It targets the spike protein of 2019-nCoV. The other vaccine, Ad5-nCoV (a recombinant novel coronavirus disease vaccine (adenovirus type 5 vector)), was conducted at Tongji Hospital in Wuhan, China, on March 16, 2020 [238]. The trial was jointly developed and administered by CanSino Biologics Inc. and the Academy of Military Medical Sciences. Ad5-nCoV, a genetically engineered vaccine, expresses the 2019-nCoV S protein using replication-defective adenovirus type 5 as an expression vector, thereby inducing a virus-specific immune response to prevent COVID-19. There are also several other types of COVID-19 vaccines, including deoptimized live attenuated vaccines, protein vaccine, DNA vaccine, RNA vaccine, and subunit vaccine, all of which are in the preclinical stage (Table 4) [239]. Notably, there is a new microneedle array (MNA) approach based on delivering coronavirus S1 subunit vaccines against COVID-19. Expedited by prior experience in developing vaccines against MERS, this approach was developed within 4 weeks and enabled long-term induction of potent virus-specific antibody responses. Significantly, the MNA work can be extended to other emerging infectious diseases. However, these will require further clinical studies for efficacy and safety, which requires more time.

According to the 7^th^ edition of the diagnostic criteria [25], patients severely or critically ill with COVID-19 should receive comprehensive antiviral treatment, including lopinavir/ritonavir, arbidol, or Shufeng Jiedu Capsule. Meanwhile, they also need additional treatments, according to their symptoms, including respiratory support (oxygen therapy, high-flow nasal cannulas, or noninvasive ventilation, invasive mechanical ventilation, or extracorporeal membrane oxygenation- (ECMO-) based therapy), circulatory support, or continuous renal replacement therapy. The main therapeutic approaches proposed for COVID-19 are summarized in Table 5.

## 4. Control and Prevention of COVID-19

As the most recent pandemic, COVID-19 induces much fear. It is highly infectious and is transmitted asymptomatically. As such, our best options to slow and prevent transmission are to understand the origin of 2019-nCoV, its transmission route, and associated disease pathways and systems. Generally, a pathogen must remain viable outside the host to allow for environmental spread [240]. Collective effects of many biotic and abiotic factors determine the period that the pathogen can survive [240]. As of now, COVID-19 is thought to be transmitted directly from person-to-person through liquid (droplets) and, more importantly, transmitted indirectly via contact with contaminated surfaces. 2019-nCoV remains viable for a fairly long period outside the human body (up to 72 hours) and is more stable on plastic and stainless steel than on copper and cardboard [241]. Therefore, aerosol and fomite transmission of 2019-nCoV is possible, as the virus lingers among particles or fibers, in airborne liquid droplets, and on surfaces, in some cases for days [241]. Although there are currently insufficient data on the inactivation of environmental 2019-nCoV, data from other coronaviruses can be used as a reference. However, it should be noted that biocidal agents may only limit the survival of coronavirus in critical environments and have no efficacy for infected patients.

**Figure 10:**
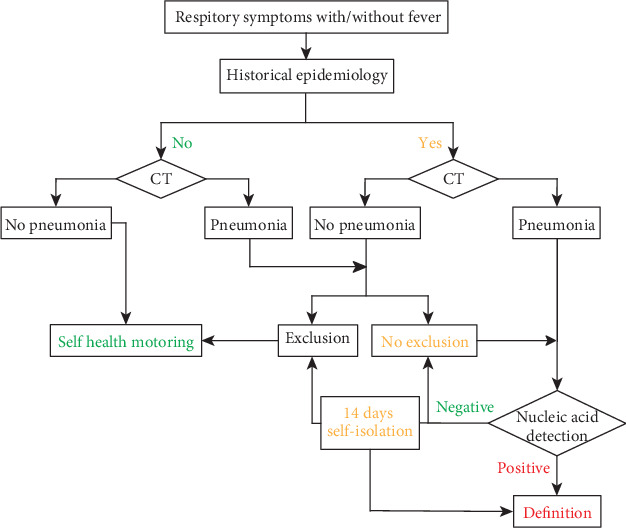
Procedures for identifying COVID-19 cases.

Given the high transmissibility of COVID-19, its propensity for asymptomatic transmission, and its persistent nature, confirmed patients could only be quarantined and treated in adequately equipped facilities. This also applies to anyone who has come into contact with these patients. As such, contact tracing is still a mainstay for disease control. Confirmation can be achieved only when specific diagnostic methods have been employed. Chest CT imaging is useful as an initial evaluation for COVID-19, as CT confirmation is often possible even before symptoms appear; therefore, it is recommended for suspected COVID-19 cases [242]. Once the primary diagnosis reveals abnormal chest CT findings, a nucleic acid test should be performed to confirm whether a patient is infected. Once a person is confirmed positive, tests such as C-reactive protein (CRP), complete blood count, urinalysis, biochemical indicators (i.e., liver enzymes, myocardial enzymes, and renal function), blood coagulation function, arterial blood gas analysis, and cytokine levels should be performed to monitor the patient's condition [189]. Chest CT should be performed as a follow-up to treatment as well [242]. The currently adopted procedure in identifying potential COVID-19 cases in China is summarized in Figure 10.

On a community scale and beyond, strict controls over human traffic are essential to limiting disease transmission. By establishing lockdowns, China has been able to bring the crisis under control. Other nations are now following the Chinese's approach in restricting movement of residents within their borders. As evidenced globally, social distancing is essential to halt the spread of COVID-19. On the other hand, individuals have the responsibilities to follow the guidelines given by the authorities, to practice good hygiene, and to behave responsibly. COVID-19, like the past epidemics, does not recognize political boundaries, ethnicity, or gender. The disease has challenged the economic and medical infrastructure of the entire globe. As evidenced by events of the past few months, the impact of the outbreak depends upon how well we are prepared to face such a challenge. Only with time will we be able to fully evaluate the measures that are being taken against COVID-19 today.

## 5. Conclusions

Previous coronavirus epidemics like SARS and MERS have expedited the process of finding useful diagnostic and therapies against 2019-nCoV. It is of paramount importance for all countries to share essential information about 2019-nCoV to mitigate its spread. Because of this strategic approach, research has been mobilized to rapidly develop diagnostic methods and worldwide implementation to minimize the impact of the pandemic. Practical diagnostic tests have aided management and contact tracing of COVID-19 cases in hotspot areas. In this regard, molecular virological techniques have assisted the scientific community in characterizing infectious agents for years. These include qRT-PCR, isothermal amplification, and CRISPR technology.

On the other hand, serological assays for antibodies and antigens present essential tools to obtain valuable information about prior exposure to 2019-nCoV and the prevalence of infection. These include ELISA and LFA technologies. Serological screening also enables novel vaccines to be assessed and supports the design of functional vaccine approaches. Other approaches, including chest computed tomography (CT) and transmission electron microscopy (TEM), can boost existing detection approaches. Notably, there has been a marked increase in the use of both CT and TEM to detect 2019-nCoV and other coronaviruses. These complimentary tools reveal the progression of suspected infection, which cannot be accomplished by conventional diagnostic means. Nonetheless, there is a pressing need for continuous development of rapid, accurate diagnostic devices and strategies to characterize unknown respiratory pathogens.

Despite these signs of progress, the present data suggest that current public health policies and improved diagnostic measures alone may not be sufficient to eradicate COVID-19 in the short term. Efficacious and novel treatments are desperately required. Presently, large numbers of ongoing clinical trials of various drugs may succeed in minimizing morbidity and mortality. We have highlighted several of them in this review. Some are highly promising, while others may require more time to demonstrate usefulness. While some drug candidates appear promising and have been used in treating COVID-19 patients in desperation, it does not necessarily mean that they are proven safe and efficacious in the long run. As such, stringent criteria must be established by health regulatory agencies. However, in the long term, vaccines and prophylactics may be required to curb the spread of 2019-nCoV.

## Figures and Tables

**Table 1:** Detection methods of 2019-nCoV classified by protein and gene targets.

Method	Sampling methods	Detection time	Accuracy/limit of detection	Advantages					Remarks and Ref.
				Rapid	Low cost	Sensitive	Portable	Visual analysis	
Antibody									
IgG/IgM-LFA	Blood	15 min	92.69%	✔	✔	✔	✔	✔	Inapplicable to early-stage detection [243]
IgG/IgM-AIE-QD	Blood	15 min	75%	✔	✔		✔	✔	Inapplicable to early-stage detection [244]
IgM-colloidal gold-LFA	Blood	15 min	Not reported	✔	✔		✔	✔	Inapplicable to early-stage detection [245]
IgG/IgM-ELISA or LFA	Blood	29 min	93.1%	✔	✔	✔	✔	✔	Inapplicable to early-stage detection [246]
IgG/IgM-colloidal gold-FLA	Blood	15-30 min	90%	✔	✔	✔	✔	✔	Inapplicable to early-stage detection^&^
Nucleic acid (whole genome)									
Nanopore target sequencing	Throat swab	6-10 h	>95%, 10 copies/mL			✔			Monitor mutation but time-consuming and costly [28]
Nucleic acid (N and E genes or ORF*1ab*/RdRp/S)									
Real-time RT-PCR	Nasopharyngeal or oropharyngeal swab	240-360 min	67%, 3.2 copies/*μ*L	N/A	N/A	N/A	N/A	N/A	Established standard method but time-consuming and requiring skilled personnel^&^
RT-LAMP	Nasopharyngeal or oropharyngeal swab	30 min	>95%				✔		A single, simple protocol but with noisy signals^&^
Close-tube Penn-RAMP (LAMP+RPA)	Synthetic RNA	100 min	7 copies per reaction	✔		✔			Suitable for home screening but requiring clinical validation [45]
Cepat	Nasopharyngeal or oropharyngeal swab	5-10 min	99%	✔		✔	✔		High specificity but requiring clinical validation [247]
CRISPR-Cas12a/RT-LAMP (DETECTR)	Nasopharyngeal/oropharyngeal swab	45 min	10 copies/*μ*L input	✔	✔	✔			Requiring clinical validation [248]
HTX COVID-19 test kit	Nasopharyngeal or oropharyngeal swab	180 min	99%			✔			Severity evaluation possible but requiring clinical validation [249]
enVision	Synthetic RNA	30 min	Not known	✔			✔		Requiring clinical validation [76]
CRISPR-Cas13a/RPA-LFA (SHERLOCK)	Synthetic RNA	<60 min	10 copies/*μ*L input	✔				✔	Simple but requiring clinical validation [60]
CRISPR-Cas12a/RT-RPA (DETECTR)	Synthetic RNA	<60 min	10 copies/*μ*L input	✔	✔	✔			Requiring clinical validation [62]
Multiparameter-chip/RPA	Synthetic RNA	<60 min	>90%	✔	✔			✔	Requiring clinical validation [250]

N/A: not available (an established method used for comparison in this table); &: commercial kits; LAMP: loop-mediated isothermal amplification; RPA: recombinase polymerase amplification; FLA: lateral flow assay; CRISPR: clustered regularly interspaced short palindromic repeats; DETECTR: DNA endonuclease-targeted CRISPR trans reporter.

**Table 2:** Potential antiviral drugs for COVID-19.

Potential therapeutic agents	Target of inhibition	Indication/purposes	Preliminary studies	Application for COVID-19	
				Case studies	Remarks
Umifenovir	Entry receptor	Antiviral drug on influenza; not yet tested for coronaviruses	N/A	Compared with favipiravir (see favipiravir)	Currently being evaluated in China
Chloroquine, hydroxychloroquine	Entry receptor	Antiviral drug on malaria; not yet tested for coronaviruses	*In vitro* antiviral activities against 2019-nCoV on primate cells [135, 138]	Ongoing	Currently being evaluated in China and the United States
Camostat mesylate	Host protease	Antiviral drug on pancreatic diseases; not yet tested for coronaviruses	*In vitro* antiviral activities against 2019-nCoV in human lung cancer cells [113]	Not known	None
Lopinavir-ritonavir	Viral protease	Used in combination with ribavirin for SARS and MERS	N/A	199 hospitalized patients, Wuhan, China (99 lopinavir+ritonavir+100 standard care) [161]	Currently being evaluated in China and the United States. However, found to be ineffective based on preliminary findings
Darunavir	Viral protease	Antiretroviral drug; not yet tested for coronaviruses	N/A	Not known	None
Ribavirin	Genome replication	Used in combination with lopinavir-ritonavir for SARS and MERS	N/A	Not known	Currently being evaluated in China
Favipiravir	Genome replication	Antiviral drug on influenza; not yet tested for coronaviruses	N/A	240 patients in Hubei province, China (120 favipiravir+120 arbidol) (preprint) [184]	Higher recovery rate compared to those treated with umifenovir (arbidol)
Remdesivir	Genome replication	The new antiviral drug initially developed for Ebola	*In vitro* antiviral activities toward 2019-nCoV on primate cells [138]	Ongoing	Under clinical trials
Carolacton	Genome replication	Potential antibacterial compound against biofilm formation of *Streptococcus mutans* and growth of *Streptococcus pneumoniae* [192, 193]	*In vitro* antiviral activities against bat kidney cells [190]	Not known	None
Ivermectin	Genome replication	Antiparasitic drug (broad-spectrum).	*In vitro* antiviral activities against 2019-nCoV on primate cells [197]	Not known	None

N/A: not available. Note: “coronaviruses” only target SARS-CoV and MERS-CoV.

**Table 3:** TCM recommendations by guidelines for diagnosis and treatment of COVID-19 (7^th^ edition) [25].

Symptoms	Potential TCM	Main ingredients (common names)	Active ingredients	Precaution
Suspected cases				
Fatigue, gastrointestinal discomfort	Huo Xiang Zheng Qi Capsule (藿香正气胶囊)	Patchouli, Indian buead, areca peel, perilla leaf, dahurian angelica root, tangerine peel, platycodon root, largehead Atractylodes rhizome, magnolia bark, pinellia rhizome, licorice	N/A	May cause an autoimmune response. Allergic individuals are prohibited from the treatment. Patients with diseases, i.e., cardiac diseases, liver diseases, kidney diseases, hypertension, and diabetes, and pregnant women should speak to physicians before the treatment
Fatigue, fever	Lian Hua Qing Wen Capsule (连花清瘟胶囊)	Forsythia, honeysuckle flower, ephedra, apricot, gypsum, indigowoad root, crown wood-fern, heartleaf houttuynia, patchouli, rhubarb, arctic root, mint, licorice	N/A	May cause an autoimmune response. Not suitable for people with a cold. Not suitable for long-term use. Allergic individuals are prohibited from the treatment. Patients with diseases, i.e., cardiac diseases, liver diseases, kidney diseases, hypertension, and diabetes, and pregnant women should speak to physicians before the treatment
	Shu Feng Jie Du Capsule (疏风解毒胶囊)	Bushy knotweed root, forsythia, indigowoad root, thorowax root, patrinia, verbena leaf, reed root, licorice	N/A	May cause an autoimmune response, i.e., nausea. Allergic individuals and individuals with allergic constitution are prohibited from the treatment
	Jin Hua Qing Gan Pill (金花清感丸)	Honeysuckle flower, gypsum, ephedra, apricot, baical skullcap root, forsythia, fritillaria bulb, anemarrhena rhizome, great burdock achene, wormwood mint, licorice	N/A	Side effects are not known. Pregnant women, allergic individuals, and individuals with allergic constitution are prohibited from the treatment
Confirmed cases				
Mild/general/severe	Qing Fei Pai Du Decoction (清肺排毒汤)	Ephedra, licorice, apricot, gypsum, cinnamon twig, water plantain rhizome, polyporus, largehead Atractylodes rhizome, Indian buead, thorowax root, baical skullcap root, pinellia rhizome, ginger, tatarian aster root, coltsfoot flower, blackberry lily rhizome, Manchurian wildginger, Chinese yam, immature bitter orange, tangerine peel, patchouli	N/A	Not recommended to be used as a precautionary measure
Severe	Xi Yan Ping Injection (喜炎平注射液)	Andrographis	Andrographolide	May cause adverse reactions, i.e., skin rash, itchiness, fever, pain, dyspnea, cyanosis, palpitations, and convulsions. Pregnant women and allergic individuals are prohibited from the treatment
	Xing Nao Jing Injection (醒脑静注射液)	Musk, borneol, cape jasmine fruit, turmeric tuber	N/A	May cause an autoimmune response, i.e., skin rash. Pregnant women are prohibited from the treatment
Severe/critical	Xue Bi Jing Injection (血必净注射液)	Safflower, peony root, Szechuan lovage rhizome, red sage root, Chinese angelica root	Hydroxysafflor yellow A	May cause an autoimmune response, i.e., itchiness. Pregnant women are prohibited from the treatment
	Re Du Ning Injection (热毒宁注射液)	Wormwood, honeysuckle flower, cape jasmine fruit	N/A	May cause an autoimmune response, i.e., dizziness, chest tightness, thirstiness, diarrhea, nausea, vomit, skin rash, and itchiness. Allergic individuals are prohibited from the treatment
	Tan Re Qing Injection (痰热清注射液)	Baical skullcap root, bear bile, goat horn, honeysuckle flower, forsythia	N/A	May cause an autoimmune response, i.e., dizziness, nausea, vomit, itchiness, and skin rash
Critical	Shen Fu Injection (参附注射液)	Red ginseng, Chinese aconite	N/A	May cause an autoimmune response, i.e., tachycardia, skin rash, dizziness, headache, hiccup, tremor, dyspnea, nausea, visual abnormality, abnormality in liver function, and urinary retention. Newborns, allergic individuals, and individuals with allergic constitution are prohibited from the treatment
	Sheng Mai Injection (生脉注射液)	Red ginseng, Ophiopogon, magnolia berry	N/A	May cause an autoimmune response, i.e., anaphylactic shock. Newborns, pregnant women, allergic individuals, and individuals with allergic constitution are prohibited from the treatment
	Shen Mai Injection (参麦注射液)	Red ginseng, Ophiopogon	N/A	May cause autoimmune response and adverse effect on treated patients, i.e., anaphylactic shock and damage to the body systems. Newborns, pregnant women, allergic individuals, and individuals with allergic constitution are prohibited from the treatment
	Su He Xiang Pill (苏合香丸)	Storax, benzoin, borneol, musk, buffalo horn, sandalwood, agarwood, clove, nut grass, costus root, frankincense, long pepper fruit, largehead Atractylodes rhizome, gall nut, Cinnabaris	N/A	Side effects are not known. Pregnant women are prohibited from the treatment
	An Gong Niu Huang Pill (安宫牛黄丸)	Buffalo horn, musk, pearl, realgar, coptis root, Cinnabaris, baical skullcap root, cape jasmine fruit, turmeric tuber, borneol	N/A	May cause autoimmune responses, i.e., hypothermia. Prescription guidance is unclear

N/A: not available.

**Table 4:** Summary of 2019-nCoV vaccine developments at different stages [239].

Vaccine name	Vaccine type/platform	Developer	Vaccine name	Vaccine type/platform	Developer
Ad5-nCoV^∗∗^	DNA/adenovirus type 5 vector	CanSino Biological Inc., Beijing Institute of Biotechnology	Vaxil Bio COVID-19 vaccine	Protein subunit/signal peptide combinations	Vaxil Bio
INO-4800	DNA/DNA plasmid, electroporation device	Inovio Pharmaceuticals	COVID-19S-Trimer	Protein subunit/pandemic adjuvant system	Clover Biopharmaceuticals Inc./GSK
N/A^∗^	DNA/S gene	Takis/Applied DNA Sciences/Evvivax	N/A	Protein subunit/S protein	AJ Vaccine
N/A	DNA plasmid	Zydus Cadila	N/A	Protein subunit/li-Key peptide	Generex/EpiVax
N/A	Ad26/MVA boost	Janssen Pharmaceutical Companies	N/A	Protein subunit/S protein	EpiVax/Univ. of Georgia
GV-MVA-VLP/vaccine	DNA/nonreplicating viral vector	GeoVax/BravoVax		Protein subunit/S protein (baculovirus production)	Sanofi Pasteur
ChAdOx-nCoV-19^∗^	DNA/nonreplicating viral vector	University of Oxford	N/A^∗^	Protein subunit/Full-length protein Matrix-MTM adjuvant	Novavax
N/A	DNA/nonreplicating viral vector, NasoVAX-S gene	Altimmune	Heat's gp96 based vaccine for COVID-19	Protein subunit/Heat's gp96 backbone to express antigen of COVID-19	Heat Biologics/Univ. of Miami
N/A	DNA/nonreplicating viral vector (Ad5 S)	Greffex	N/A	Protein subunit/molecular clamp stabilized spike protein	University of Queensland/GSK
N/A	DNA/nonreplicating viral vector (VAAST); oral vaccine platform	Vaxart, Inc.	N/A	Protein subunit/molecular clamp stabilized spike protein	University of Queensland/GSK
N/A	DNA vaccine/measles vector (replicating viral vector)	Vaxart, Inc.	N/A	Protein subunit/S1 or RBD protein	Baylor College of Medicine
N/A	DNA vaccine/measles vector	Institute Pasteur/Themis/Univ. of Pittsburg Center for Vaccine Research	N/A	Protein subunit/plant-based coronavirus	iBio/CC-Pharming
TNX-1800/COVID-19	DNA vaccine/horsepox vaccine platform-S gene	Tonix Pharma/Southern Research	N/A^∗^	Protein subunit/protein	VIDO-InterVac, University of Saskatchewan
mRNA-1273^∗∗^	mRNA/novel lipid nanoparticle	Moderna/NIAID	N/A	Protein subunit/protein	University of Saskatchewan
N/A	mRNA vaccine/VLP-cocktail (S, M, E, N)/lipid nanoparticle	Fudan University/Shanghai JiaoTong University/RNACure Biopharma	Live attenuated virus^∗^	Deoptimized live attenuated vaccines	Codagenix/Serum Institute of India
N/A	mRNA vaccine/lipid nanoparticle/S and S-RBD antigen	Fudan University/Shanghai JiaoTong University/RNACure Biopharma	VLP Vaccine	Plant-derived VLP/four structural of rotavirus (VP2, VP4, VP6, and VP7)	Medicago Inc.
N/A	mRNAvaccine/LUNAR®-nanoparticle nonviral delivery system	Arcturus/Duke-NUS	Inactivated Vaccine	Formalin-inactivated+alum adjuvanted	Sinovac
BNT162^∗^	mRNA vaccine	BioNTech/Fosun Pharma/Pfizer	N/A	Unknown	University of Hong Kong
N/A	Self-amplifying RNA vaccine	Imperial College London	N/A	Unknown/IPA's proprietary discovery platforms	ImmunoPrecise
N/A	mRNA	CureVac	N/A	Unknown	MIGAL Galilee Research Institute
N/A	Protein subunit/Drosophila S2 insect cell expression system VLPs	ExpreS2ion	N/A	Unknown	Doherty Institute
N/A	Protein/S protein	WRAIR/USAMRIID	N/A	Unknown	Tulane University

^∗∗^In clinical studies. ^∗^Preparing for clinical studies. N/A: not available.

**Table 5:** Summary of main therapeutic approaches proposed for COVID-19.

Drug names	Original indication	Mechanism of action	Clinical status		References
Chemical drugs					
Umifenovir	Influenza A & B	Inhibition of binding of the virus to host cell membrane	Phase 4 trials for COVID-19	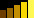	[122–124]
Chloroquine and derivatives	Malaria	Inhibition of lysosomal activity and signaling pathway in virus	Phase 2-3 trials for prophylaxis of COVID-19 Phase 2-4 trials for the treatment of COVID-19	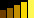	[130–140]
Lopinavir	HIV	Proposed inhibition of 3CLpro	Phase 2-4 trials for the treatment of COVID-19	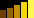	[159, 160]
Ritonavir	HIV	Proposed inhibition of 3CLpro	Phase 2-4 trials for the treatment of COVID-19	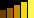	[159, 160]
Darunavir	HIV	Inhibition of viral maturation pathways	Phase 2-3 trials for the treatment of pneumonia caused by COVID-19	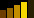	[164]
Camostat mesylate	Pancreatic inflammation	Inhibition of viral entry into host cells	Preclinical	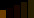	[113, 151, 152]
Remdesivir	Ebola (proposed)	Inhibits viral RNA polymerase	Phase 3 trials for the treatment of COVID-19	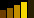	[186, 188]
Favipiravir	Influenza	Inhibits RdRp	Phase 3 and randomized trials for the treatment of COVID-19	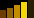	[138, 179–182]
Ribavirin	HCV, RSV	Inhibition of viral RNA replication & increased mutation in viral RNA	Phase 2 trials for the treatment of COVID-19	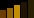	[168–172]
Biologics					
INF-*α*	HCV, HCL, melanoma	Invoking interferon response through exogenous interferons	Multiple randomized trials for COVID-19 patients	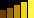	[208, 209]
Convalescent plasma	Influenza, Ebola, SARS, MERS	Suppression of viremia	Multiple randomized trials for COVID-19	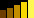	[212, 213]
Human monoclonal antibodies	Nil	Binding to the receptor-binding domain of COVID-2019	Preclinical	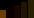	[214]
mRNA-1273, Ad5-nCoV	Nil	Eliciting immune response via host cell-expressed viral protein	Phase 1 clinical trials for prevention of COVID-19 infection	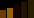	[237, 238]
B & T cell epitopes	SARS-CoV	Invoke immune targeting of these epitopes	Preclinical	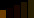	[251]
MSC	Heart disease, Parkinson's disease, lung cancer, type 1 diabetes, stroke	Promotes endothelial repair and reduce inflammation through secretion of soluble paracrine factors	Phase 0-1 trials for the treatment of COVID-19	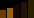	[235, 236]
TCM/herbal remedies					
Lian Hua Qing Wen Capsule	Influenza	Downregulates MCP-1 which decreases monocytes chemotaxis to infection foci	Phase 4 trials for the treatment of COVID-19	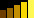	[25, 252]
Xue Bi Jing Injection	Inflammation	Inhibition of proinflammatory Th17 cells and reduced inflammatory cytokines TNF-*α* and IL-6	Phase 0-4 trials for the treatment of COVID-19	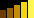	[25, 232, 253]
Re Du Ning Injection	URTI	Suppressing secretion of inflammatory mediators	Phase 0 trial for the treatment of COVID-19	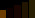	[25, 254]
Xi Yan Ping Injection	HFMD, URTI	Inhibition of NF-*κ*B and MAPK-mediated inflammatory responses	Phase 0 trial for the treatment of COVID-19	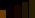	[25, 255]
Tan Re Qing Injection/Capsule	URTI, COPD	Inhibition of NF-*κ*B and MAPK-mediated inflammatory responses	Phase 0-4 trials for the treatment of COVID-19	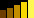	[25, 256]
Shen Fu Injection	Congestive heart failure, ischemic stroke	Affect various immune signaling pathways to have a protective effect against organ damage	Phase 4 trial for the treatment of COVID-19	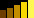	[25, 257]

Abbreviations: 3CLpro: 3C-like protease; COPD: chronic obstructive pulmonary disease; HCL: hairy cell leukemia; HCV: hepatitis C virus; HFMD: hand, foot, and mouth disease; HIV: human immunodeficiency virus; IL-6: interleukin 6; INF-*α*: interferon-alpha; MAPK: mitogen-activated protein kinase; MCP-1: monocyte chemoattractant protein-1; MERS-CoV: Middle East respiratory syndrome-coronavirus; MSC: mesenchymal stem cell; NF-*κ*B: nuclear factor kappa-light-chain-enhancer of activated B cells; RdRp: RNA-dependent RNA polymerase; RNA: ribonucleic acid; RSV: respiratory syncytial virus; SARS-CoV: severe acute respiratory syndrome-coronavirus; Th17: T helper 17; TNF-*α*: tumor necrosis factor-alpha; URTI: upper respiratory tract infection.
